# The Greatwall kinase safeguards the genome integrity by affecting the kinome activity in mitosis

**DOI:** 10.1038/s41388-020-01470-1

**Published:** 2020-09-25

**Authors:** Xavier Bisteau, Joann Lee, Vinayaka Srinivas, Joanna H. S. Lee, Joanna Niska-Blakie, Gifford Tan, Shannon Y. X. Yap, Kevin W. Hom, Cheng Kit Wong, Jeongjun Chae, Loo Chien Wang, Jinho Kim, Giulia Rancati, Radoslaw M. Sobota, Chris S. H. Tan, Philipp Kaldis

**Affiliations:** 1 Institute of Molecular and Cell Biology (IMCB), A*STAR (Agency for Science, Technology and Research), Singapore, 138673 Republic of Singapore; 2 Program in Cancer and Stem Cell Biology, Duke-NUS Graduate Medical School, Singapore, 169857 Republic of Singapore; 3 Bioinformatics Institute (BII), A*STAR, Singapore, 138671 Republic of Singapore; 4 Institute of Medical Biology, A*STAR, Singapore, 138648 Republic of Singapore; 5 Department of Health Sciences and Technology, SAIHST, Sungkyunkwan University, Seoul, 06351 Korea; 6 Functional Proteomics Laboratory, IMCB, A*STAR, Singapore, 138673 Republic of Singapore; 7 Samsung Genome Institute, Samsung Medical Center, Gangnam-gu, Seoul, 06351 Korea; 8 Department of Clinical Sciences, Clinical Research Centre (CRC), Lund University, Box 50332, 202 13 Malmö, Sweden; 9 Present Address: IRIBHM—Université Libre de Bruxelles ULB, Campus Erasme Blg C, Brussels, Belgium; 10 Present Address: Ewha Woman’s University, 52 Ewhayeodae-gil, Daehyeon-dong, Seodaemun-gu, Seoul Republic of Korea; 11 Present Address: Department of Chemistry, College of Science, Southern University of Science and Technology, Shenzhen, China

**Keywords:** Experimental organisms, Predictive markers

## Abstract

Progression through mitosis is balanced by the timely regulation of phosphorylation and dephosphorylation events ensuring the correct segregation of chromosomes before cytokinesis. This balance is regulated by the opposing actions of CDK1 and PP2A, as well as the Greatwall kinase/MASTL. MASTL is commonly overexpressed in cancer, which makes it a potential therapeutic anticancer target. Loss of *Mastl* induces multiple chromosomal errors that lead to the accumulation of micronuclei and multilobulated cells in mitosis. Our analyses revealed that loss of *Mastl* leads to chromosome breaks and abnormalities impairing correct segregation. Phospho-proteomic data for *Mastl* knockout cells revealed alterations in proteins implicated in multiple processes during mitosis including double-strand DNA damage repair. In silico prediction of the kinases with affected activity unveiled NEK2 to be regulated in the absence of *Mastl*. We uncovered that, RAD51AP1, involved in regulation of homologous recombination, is phosphorylated by NEK2 and CDK1 but also efficiently dephosphorylated by PP2A/B55. Our results suggest that *MastlKO* disturbs the equilibrium of the mitotic phosphoproteome that leads to the disruption of DNA damage repair and triggers an accumulation of chromosome breaks even in noncancerous cells.

## Introduction

The progression of a proliferating cell through the cell cycle, mitosis, and finally its division is finely regulated by the balance of multiple phosphorylation and dephosphorylation events. This dynamics is achieved by the competing actions of kinases and phosphatases. Cyclin-dependent kinases (CDKs) drive the progression through the cell cycle [1–6]. CDK1/cyclin B1 complexes initiates mitotic entry by phosphorylating a multitude of proteins to condense chromosomes, disrupt the nuclear envelope, and enable microtubules polymerization to attach and to segregate the chromosomes. All those phosphorylation events need to be reversed once cells exit mitosis and the entire phosphoproteome must be reset for the next cell cycle. A group of phosphatases has been reported to reverse the action of these kinases and regulate the precise timing of mitotic exit [7]. The Greatwall kinase, called MASTL in mammals, has been shown to enhance mitotic entry by phosphorylating two small proteins, ARPP19/ENSA, that bind and inhibit the PP2A/B55 complex, therefore preventing the dephosphorylation of substrates at the entry of mitosis [8–10]. To ensure the metaphase–anaphase transition and subsequent mitotic exit, cyclin B1 is ubiquitinated by APC^Cdc20^ to promote its degradation, triggering a drop in CDK1 activity [11]. Following the decreased activity of CDK1, inhibition of PP1 is relieved, triggering FCP1 activation, which in turn dephosphorylates MASTL. Similarly, the increasing activity of PP1 enhances the activation of PP2A/B55 [7, 12–15]. This creates a bistable switch to exit mitosis [16, 17]. Altering the MASTL-ENSA/ARPP19-PP2A/B55 pathway induces numerous mitotic errors during chromosome segregation and cytokinesis that can drive further chromosomal instability and consequently tumorigenesis [18–21]. Because MASTL is essential during mitosis, this pathway is considered as a potential anticancer therapeutic target. A growing number of studies have reported that *Mastl* deficiency in multiple cancers like breast cancer, thyroid cancer, and leukemia reduced cellular proliferation and/or tumor size [22–26]. Similarly, chemical reactivation of PP2A in some cancers resulted in regression of the tumor to some extent [27–29]. Interestingly, MASTL is overexpressed in several cancers with an associated increase of chromosome instability and associated with a poorer outcome of patients [25, 30]. This indicates that the balance and the precise timing of the phosphorylation events during mitosis are crucial to ensure correct chromosome segregation and mitosis. In this context, it would be benefical to decipher how the MASTL pathway affects global phosphorylation events as well as how this affects the kinome, which drives mitotic progression. Here we used extensive phosphoproteomics to compare the difference in phosphorylation and dephosphorylation events between WT and *MastlKO* MEFs. Our results suggest that the lack of *Mastl* induces chromosomal errors well before the metaphase–anaphase transition by affecting not only mitotic processes but also many other kinases.

## Results

### Loss of *Mastl* induces micronuclei

Previous reports employed several methodologies and systems to delete the Greatwall kinase [10, 18, 19, 21, 22, 25, 31]. We opted here to use our recently developed inducible knockout model to ablate *Mastl* with near complete penetrance in all cells [21]. Using this system where immortalized mouse embryonic fibroblasts (MEFs) are treated with 4-hydroxytamoxifen (4-OHT) to induce *Mastl* deletion, we observed in asynchronous culture the appearance of micronuclei in 76% of cells within 24 h (Fig. S1A) post treatment. This proportion continued to increase, reaching 90% of the fibroblasts deprived of *Mastl* (Fig. S1B) and led to the formation of multinucleated and multilobulated cells (Fig. S1C) as previously reported [21]. This abrupt increase of cells with micronuclei at 24 h correlates with the time cells take to progress into mitosis and complete one cell cycle (24–28 h). By synchronizing cells in quiescence and releasing them into the cell cycle by the re-addition of serum, we further confirmed this correlation. The proportion of cells with micronuclei rises drastically at 40 h post release (Fig. S1D; 54%) and continued to increase at 48 h (65%). This contrasted with the slow increase of cells displaying micronuclei starting at 6 (Fig. S1D, 6%), 16 (10%), and 24 h (13%) after release. In parallel, the number of micronuclei per cells accumulated over time (Fig. S1E). This indicated that in G1 (6h) and S phase (16h), only few *Mastl*^*NULL*^ cells displayed micronuclei and these only had 1–2 micronuclei. At 24 h, the few *Mastl*^*NULL*^ MEFs with micronuclei displayed more than two micronuclei (Fig. S1E). This accumulation further continued at 40- and 48-h post release, indicating that as time progressed the phenotype became more prominent. Therefore, we hypothesized that micronuclei formed as cell progressed through mitosis.

#### Non-congressed chromosomal fragments in *Mastl*^*NULL*^ cells

**Fig. 1:**
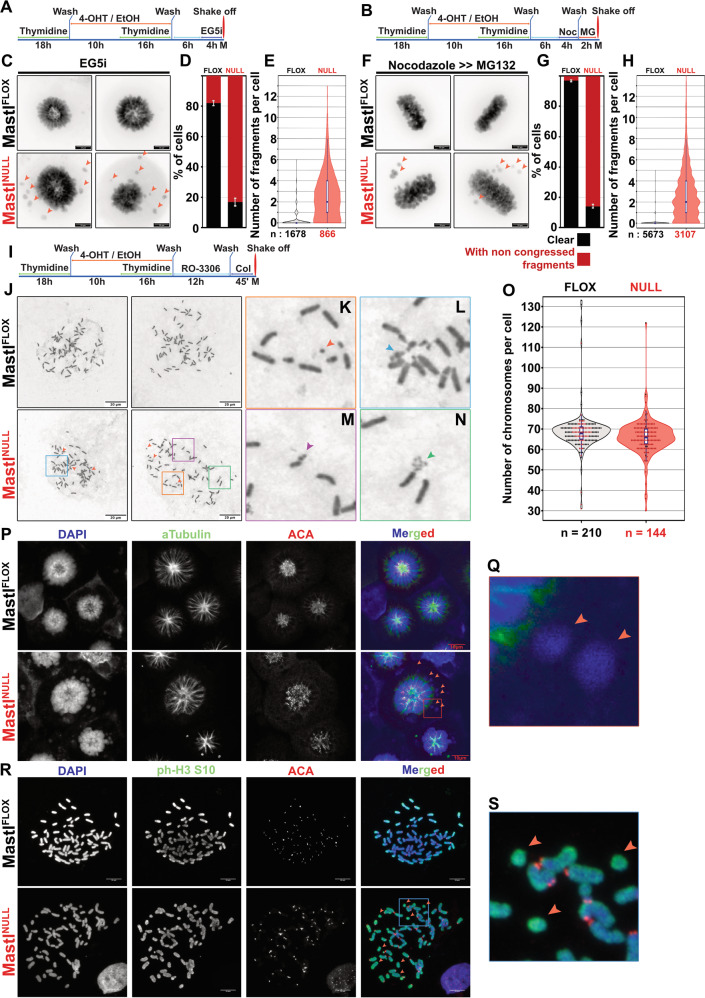
The loss of *Mastl* induces chromosomal rearrangements and fragmentation. Cartoon of the approach and timing used to synchronize cells in specific cell-cycle phases by double thymidine block before collecting mitosis-arrested cells by mitotic poisons EG5i (**a**) or Nocodazole [Noc]>>MG132 [MG] (**b**) via mitotic shake-off. **c**, **f** Representative images of Hoechst-stained *Mastl*^*FLOX*^ and *Mastl*^*NULL*^ iMEFs arrested in mitosis with **(c)** Eg5 inhibitor or with (**f)** Nocodazole>>MG132 (*N* = 3). Orange arrows indicate non-congressed fragments. **d**, **g** Percentage of cells from **c** and **f** with or without fragments, respectively. **e**, **h** Violin plots with box plot of the distribution of non-congressed fragments per cell from **c** and **f**, respectively. **i** Cartoon of the approach and timing used to synchronize cells by double thymidine block followed by a block in G2/M transition by RO-3306 CDK1 inhibitor before collecting mitosis-arrested cells by mitotic poisons colcemid via mitotic shake-off for chromosome spreads analyses. **j** Representative images of Giemsa-stained chromosomes spread from *Mastl*^*FLOX*^ and *Mastl*^*NULL*^ iMEFs. Orange arrows indicate chromosomal fragments (*N* = 2). Magnified images of fragments (**k**) and aberrant chromosomes from *Mastl*^*NULL*^ iMEFs (**l**–**n**). **o** Violin plot with embedded box plots and dot plots of the distribution of the number of chromosomes per cell from *Mastl*^*FLOX*^ and *Mastl*^*NULL*^ iMEFs with black and red dots depicting cells without or with chromosomal fragments, respectively. Representative images of immunofluorescence staining of *Mastl*^*FLO*X^ and *Mastl*^*NULL*^ iMEFs for **p** cells arrested in mitosis with Eg5i or **r** chromosomes spreads stained with DAPI, and stained with antibodies against the centromere (ACA) and the spindle (αTubulin) or phospho-Histone H3 on S10. **q**, **s** Magnified images of fragments from *Mastl*^*NULL*^ iMEFs from **p** and **r**, respectively. *n*: number of counted cells.

To decipher how the loss of the Greatwall kinase induces such strong defects, we evaluated chromosome congression in cells with or without the Greatwall kinase. Mitotic cells were collected after their synchronization using a double thymidine block (DTB) following arrest in mitosis after exposure to an inhibitor of kinesin Eg5 (EG5i) (Fig. 1a) or by a sequence of drugs (Nocodazole >> MG132) (Fig. 1b). Both treatments arrested WT cells in mitosis with normal chromosome congression. Eg5 inhibition created a monoastral ring whereas the sequence of drug blocked cells with a formed metaphase plate (Fig. 1c, f, upper panels) as expected. Although both conditions arrested cells at the stage of chromosome congression, *Mastl*^*NULL*^ cells displayed heterogenous and irregular forms of the monoastral ring and the metaphase plate. The acquired images of *Mastl*^*NULL*^ cells unveiled the appearance of numerous spots labeled by DAPI or Hoechst dyes (Fig. 1c, f, lower panels), which were not congressed with the other chromosomes. Despite that the identity of these non-congressed DNA remained elusive so far, they will be referred to as “fragments.” Although fragments could be observed in a few wild-type (WT)/*Mastl*^*FLOX*^ cells (Fig. 1d; 18.2%), their proportion in *Mastl*^*NULL*^ MEFs appeared substantially higher (83.1%). Cells arrested in metaphase with Noco/MG132 revealed the same behavior with fragments outside the metaphase plate (Fig. 1f, g; 3% for WT and 85.9% for *Mastl*^*NULL*^). The distribution of such fragments revealed a similar trend in those cells arrested with both treatments, parallel to the observation that the number of micronuclei per cells increased in absence of the Greatwall kinase, indicating that the drug treatment was not the cause for the fragments. Whereas few WT MEFs displayed up to five fragments per cell, in the *Mastl*^*NULL*^ cells, there was a heterogenous distribution of fragments [in the range of 1–20 fragments per cell] (Fig. 1e, h). Similar results were obtained using several clones of primary MEFs deficient for *Mastl* (see Fig. S2A, B), indicating that these results were not caused by the immortalization process of the cells used.

The cause(s) leading to the observed congression defect in the absence of *Mastl* as well as the identity of such fragments remain elusive. To evaluate the impact of the loss of *Mastl* on chromosomes, we prepared chromosome spreads labeled with GIEMSA (Fig. 1j) or DAPI (Fig. S2C). To avoid hypercondensation of the chromosomes due to longer exposure to demecolcine, double thymidine-blocked MEFs were further arrested at the G2/M transition by the addition of 9 µM of CDK1 inhibitor RO-3306 for 12 h (Fig. 1i). Once the inhibitor was washed out, cells were immediately blocked in mitosis by exposure to demecolcine for 45 min. This led to mitotic cells with a homogenous size of chromosomes but with remaining strong cohesion of the sister chromatids different to the normal V shape expected for mouse chromosomes (Fig. 1j, S2C). Counting of chromosomes per cell revealed that immortalized MEFs displayed around 70 chromosomes per cell (Figs. 1o and S2D; *Mastl*^*FLOX*^). The distribution of chromosomes obtained from the knockout cells appeared to be more dispersed and shifted to ~64 chromosomes per cell (Figs. 1o and S2D*; Mastl*^*NULL*^). In contrast to WT cells, a varied and heterogenous panel of chromosome defects was observed from prometaphase-arrested *Mastl*^*NULL*^ MEFs. Not only fragments (Fig. 1j, k, orange arrows) were observed but also chromosome breaks, gaps, and premature decondensation (Fig. 1l–n). Even though such defects were also observed in WT cells (Fig. S2E–H), their proportion in *Mastl*^*NULL*^ MEFs was increased (87.5% with fragments and 72.2% with chromosomal defects in *Mastl*^*NULL*^ vs. 30% and 28.1% in WT, respectively) with a median of four fragments and two chromosomal defects per cell in the absence of *Mastl* (Figs. S2F, H).

To further define the observed DAPI-labeled spots, we immunostained mitotic cells for the centromere (ACA) and the spindle [alpha-tubulin] (Fig. 1p). This revealed not only an absence of attachment of the fragments to the spindle but also the inexistence of centromere staining on the non-congressed fragments (Fig. 1p, q). Nevertheless, properly congressed chromosomes from both conditions (WT and *Mastl*^*NULL*^) displayed correct ACA staining. Chromosome spreads stained with ACA confirmed this observation (Fig. 1r). In the absence of *Mastl*, multiple small chromosomes or fragments were not stained for ACA while being fully stained for phosphorylation on S10 of histone H3 (mitotic marker) and DAPI (Fig. 1r, s).

Taken together, our results suggest that the Greatwall kinase/Mastl safeguards chromosomes integrity and its absence induces chromosomal abnormalities first observable in mitosis. The accumulation of such defects tends to create micronuclei that remain unresolved over the next cell cycle, which results in aberrant cellular ploidy, lobulation, and nucleation.

### Balanced kinase activity is essential during mitotic progression

#### Loss of *Mastl* broadly alters the phosphoproteome without affecting the proteome

**Fig. 2:**
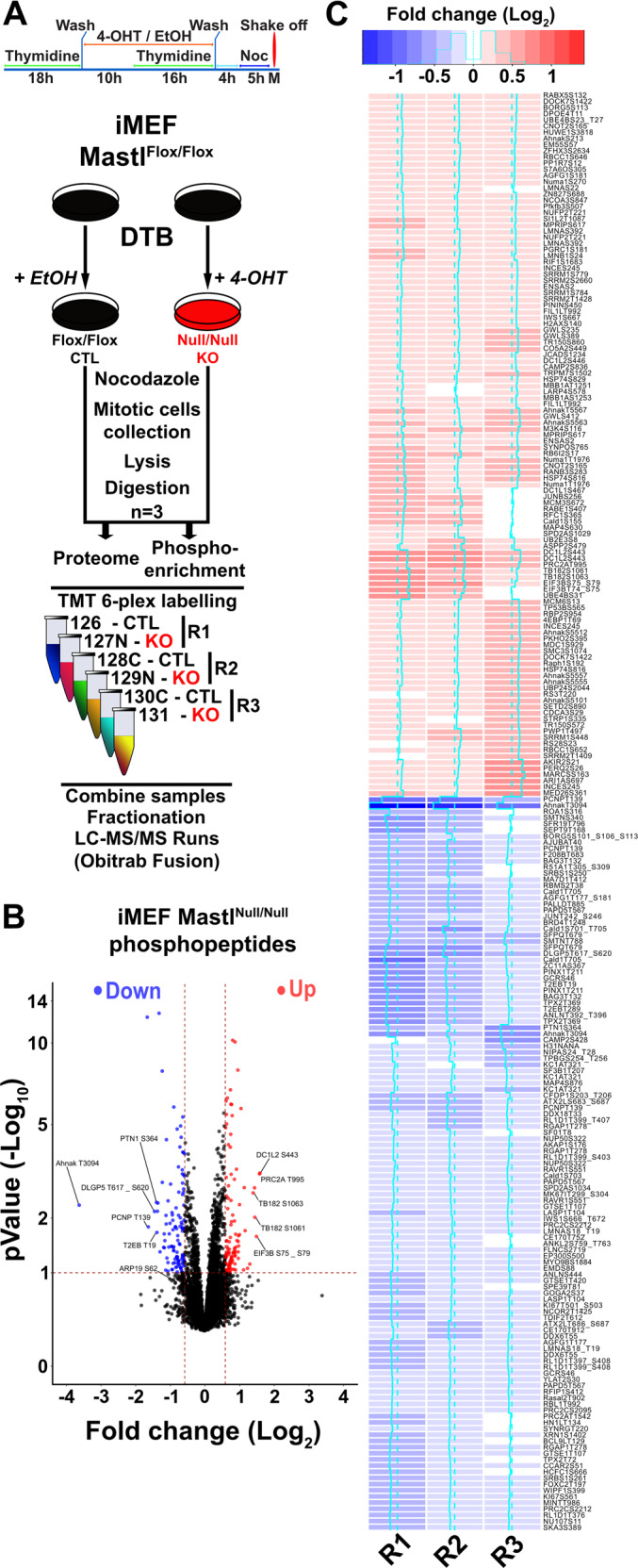
Alteration of the phosphoproteome in the absence of *Mastl*. **a** Scheme of experiment for quantitative MS-based proteomic and phospho-proteomic analyses of *Mastl*^*FLOX*^ and *Mastl*^*NULL*^ iMEFs from three paired biological replicates (*N* = 3). **b** Volcano plot of the fold-change (*Mastl*^*NULL*^/*Mastl*^*FLOX*^) vs. the pValue of the quantified phosphopeptides. Red dots: upregulated phosphopeptides (FC ≥ 1.5 and pValue ≤ −0.1). Blue dots: downregulated phosphopeptides (FC ≤ 0.667 and pValue ≤ −0.1). Labels depict the five most regulated phosphopeptides of each category. **c** Heatmap of up- and downregulated phosphopeptides.

The Greatwall kinase, through inhibition of PP2A-B55 activity, is expected to regulate a vast repertoire of phosphorylation events. However, most of those events are unknown and their effects remain elusive. In order to unveil and identify novel events directly or indirectly regulated by *Mastl* and possibly link those to the observed phenotypes, we performed mass-spectometry-based quantitative proteomic and phospho-proteomic analyses (Fig. 2a). WT and *Mastl*^*NULL*^ immortalized MEFs were synchronized by DTB, released and arrested in mitosis by addition of nocodazole before the collection of mitotic cells by mitotic shake-off (Fig. 2a). Three separate paired experiments were performed. All the collected cells were then processed in parallel as depicted in Fig. 2a. After lysis and trypsin digestion, 5% of each sample was taken for proteome analysis while the remaining portions were enriched for phosphopeptides using TiO_2_ beads. All the samples were subsequently labeled using a TMT-6 scheme (Fig. 2a), combined, and analyzed with LC-MS/MS. The proteome analysis quantified 7823 proteins across each replicate and condition. Despite the great number of quantified proteins, only MASTL displayed a significant change in expression level between WT and *Mastl*^*NULL*^ MEFs after adjustement for false discorvery rate (Fig. S3A and Table S1). This confirms the loss of the MASTL protein and indicates that the global protein levels were not affected in *Mastl*^*NULL*^ MEFs. This allowed us to solely focus on the phosphoproteome analysis in which 13,572 phosphopeptides were quantified, originating from 1844 different phosphorylated proteins (Fig. 2b and Table S2). Comparison of the overall intensity of the phosphopeptides between conditions and replicates (Fig. S3B) indicated minimal differences with a Pearson coefficient no <0.93. However, the comparison of the obtained ratio from each replicate revealed another trend (Fig. S3C, D). The distribution of the fold-change from the third replicate indicated a wider dispersion than observed for the first two replicates (Fig. S3C). Similarly, the scatterplot of the last replicate compared to the two first revealed a bigger dissimilarity with a weak Pearson correlation (Fig. S3D). Because of the divergence of the third replicate, we evaluated the significance using the reproducibility between replicates based on the ratios (Table S2, pValue.Sample) or on the MS spectra in each TMT channel (pValue.rat). The calculated pValues based on the spectra were more stringent and shed light on phosphopeptides with a higher reproducibility between the three replicates (Fig. 2c). Therefore, only this pValue (pValue.rat) was used in the following analyses unless stated otherwise. In this context, 233 phosphopeptides were considered significantly regulated with 119 downregulated (FC ≤ 0.667 and pValue ≤ 0.1) and 114 upregulated (FC ≥ 1.5 and pValue ≤ 0.1) (Fig. 2b, c), which was somewhat surprising since the loss of *Mastl* mostly hyperactivates PP2A that should result in hypophosphorylated proteins.

#### Most of mitotic and DNA double-strand break repair processes are altered after Mastl ablation

**Fig. 3:**
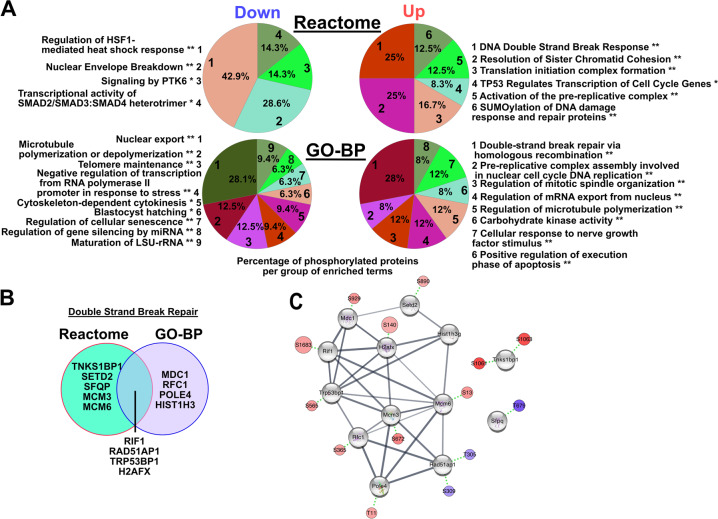
Loss of *Mastl* affects mitotic prophase and DNA repair processes. **a** Pie chart of the percentage of phosphorylated proteins associated to grouping terms from the Reactome or the biological processes from the gene ontologies (GO_BP) on up- and downregulated phosphopeptides. **P ≤ 0.001, *P ≤ 0.01. **b** Venn diagram of genes annotated to DNA double-strand break repair term in the Reactome and GO-BP database. **c** Subset of protein–protein interactions shown in File S1 for the proteins annotated to DNA double-strand break repair with regulated phosphorylation. Red represents upregulation and blue downregulation.

To understand the altered phosphorylation events and decipher how such events are possibly connected to the observed defects, we investigated the processes and pathways that were affected in the absence of *Mastl* based on the regulated phosphoproteins. Therefore, the up-and downregulated phosphoproteins were subjected to enrichment analysis using ClueGO in Cytoscape to visualize the association of the Reactome pathway terms or the gene ontology terms. Using a term grouping option to reduce the dispersion of the terms, we uncovered several enrichments as presented in Figs. 3a and S4. The enrichment grouped approximatively half of all the regulated phosphoproteins into the mitotic prophase (Fig. S4A, Table S3, and File S1). Extending this group of phosphoproteins categorized under mitotic prophase to phosphoproteins linked to cell-cycle checkpoints, cell cycle, and mitosis, with more than 70% of the phosphoproteins connected to these terms. This was not surprising as the collected cells for phosphoproteome analysis were not only arrested in mitosis but displayed defects only observable during this phase. Several of these proteins such as hypophosphorylated Lamin A/C (LmnA) and Lamin B1 (LmnB1) were grouped in the mitotic prophase on top of their role involved in “nuclear envelope breakdown” (Figs. 3a and S4A). Among the hypophosphorylated proteins, some were associated with nuclear export like Nup50 and Nup107, whereas a smaller group regulating microtubule de/polymerization was also enriched (Fig. S3A and Table S3). Surprizingly, a small group of three proteins (NIFK, DDX18, and RSL1D1) was associated with the maturation of LSU-rRNA. Inversely, several hyperphosphorylated proteins interacted with this group and were enriched for translation initiation (Fig. 3a and File S1). While being well connected to cell-cycle checkpoints, 13 phosphoproteins also formed a cluster for DNA double-strand break repair including H2AFX, 53BP1, MDC1, RFC1, RIF1, and RAD51AP1 (Figs. 3b and S4A, B) that was the most enriched cluster among the hyperphosphorylated proteins (Fig. 3a).

Our data revealed that the loss of *Mastl* alters the phosphoproteome not only by reducing the phosphorylation level of numerous proteins, directly or indirectly through PP2A/B55, but also consequently triggered an increase in phosphorylation events. Enrichment analysis unveiled that the altered phosphoproteins clustered through a network of protein–protein interactions (File S1) that affected mitosis and the cell cycle but also the DNA double-strand break repair forming a protein cluster in which only phosphorylation of RAD51AP1 and SFPQ appeared downregulated (Fig. 3c).

#### Both CDK and ATM/ATR phosphorylated motifs are regulated

**Fig. 4:**
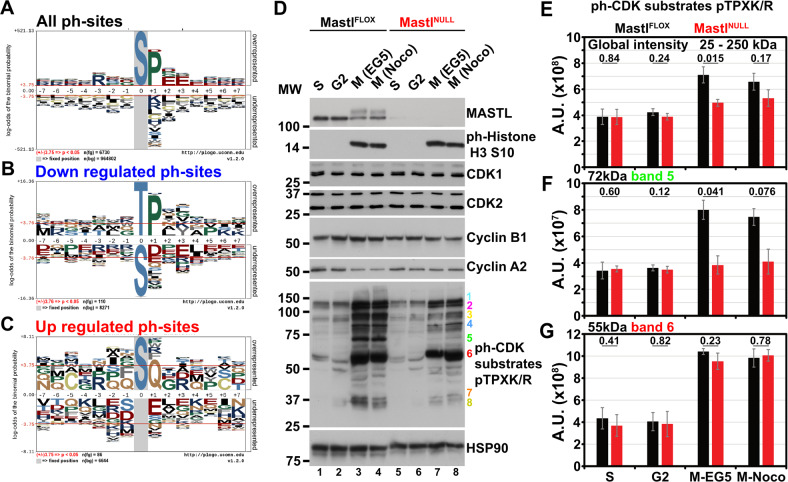
CDK phosphomotifs are unstable in the absence of *Mastl*. Motif enrichment (pLogo, [103]) for **a** all, **b** downregulated, and **c** upregulated phosphosites. **d** Gel separation of whole cell lysates from *Mastl*^*FLOX*^ or *Mastl*^*NULL*^ iMEFs collected at different cell-cycle phases (S, G2, and mitosis [M]) and immunoblotted with antibodies against the indicated proteins. MW Molecular weight ladder. Colored numbers: ph-CDK substrates pTPXK/R. Signal quantification of **e** the global signal or of **f**, **g** the indicated bands revealed by antibodies against phospho-CDK substrates depicted in **d**. A.U. Arbitrary unit. Error bars depict SD. pValue obtained from paired *t*-test on log2-transformed values from separate blots.

In order to understand the alterations caused by the absence of *Mastl*, we compared the phosphorylation events quantified in our study to those referenced in databases like PhosphoSitesPlus (PSP) and PhosphoELM (Fig. S3E). More than half of the phosphorylation events observed in our study were already identified either solely in the PSP database (59.4%) or in both (13.3%). Around one fourth of the phosphorylation events from our study (26.9%, 2366 out of 8785) were found to be novel. Most of the quantified phosphopeptides represented one (76%) or two phosphorylation events (21.5%), while a small percentage of the quantified phosphopeptides represented three or more phosphorylation events (Fig. S3F). Among all the phosphorylation events quantified in our study, 80.3% concerned a serine, 19.1% a threonine, and only a minute amount (0.6%) a tyrosine (Fig. S3G). Noticeably, half of all phosphorylation sites quantified in this study (50.5%) contained a proline residue after the phosphorylated residue (Fig. 4a). However, the downregulated phosphorylation sites revealed another pattern of enrichment in which the phosphorylated residue was dominated by a threonine (83 of 141 phosphorylation sites; 58.8%) as previously reported [32, 33] as well as a proline in +1 position of the phosphorylated residue (116 of 141 phosphorylation sites; 82.2%) (Fig. 4b). The presence of the proline in +1 position appeared drastically increased after a phosphorylated threonine (77 of 83 phosphorylation sites; 92.7%); more so than after a serine (39 of 58 phosphorylation sites; 67.2%). This enriched motif is similar to the recent report of the B55/PP2A target sites [34]. In this context, a brief comparison of the downregulated phosphorylation sites from our study with those predicted as PP2A/B55 dependent in Cundell et al. [34] revealed some similarity despite the difference of species (Table S2). The phosphorylation on T72 and T369 of TPX2, a major regulator of the mitotic spindle appeared to be a direct target of PP2A/B55. Similarly, the dephosphorylation of SFPQ on T679 (T687 in human) is dependent of PP2A/B55. Inversely, the dephosphorylation of RSL1D1 on T376 and NIFK on T299 (T279 in human) was considered independent of PP2A/B55. Motif enrichment of the upregulated phosphorylation sites unveiled a mixed pattern. Most of the phosphorylated sites were on a serine residue (99 of 117 phosphorylation sites, 84.6%) (Fig. 4c), followed by various amino acids. A remarkable enrichment for a glutamine (Q) in +1 position of the phosphorylated residue (16 of 117 phosphorylations; 13.6%) emerged. This percentage strongly contrasted with the overall proportion of this pattern ([SQ]; 3.2%) in the entire list of phosphorylation sites. It is noteworthy that this pattern is known to be phosphorylated by ATM/ATR and DNAPK kinases and that some of the phosphorylated proteins identified by us are established targets of ATM/ATR/DNAPK such as H2AX, 53BP1, and MCM6 [35–38].

In order to validate this observation, whole cell lysates from WT and *Mastl*^*NULL*^ collected at different phases of the cell cycle (for enrichment see Fig. S5A) were analyzed by immunoblotting (Fig. 4d). As previously described [21], we were not able to observe any differences on the expression level for CDK1 and CDK2. Cyclin B1 level slightly decreased in the absence of *Mastl* (Fig. 4d, lane 6–8 vs. 2–4) especially in cells collected in mitosis using both arrests, while cyclin A2 levels remained stable in *Mastl*^*NULL*^ cells. The phosphorylation of CDK substrates displaying a [pTPXK/R] motif markedly increased in mitosis when compared to the two other phases (Fig. 4d, S and G2 vs. M). Confirming the motif enrichment analysis based on downregulated phosphorylation sites, we observed that the global intensity of phosphorylated CDK substrates was reduced in the absence of *Mastl* from mitotic cells (Fig. 4d, e), which could be a result of decreased kinase activity or increased phosphatase activity. While being weakly or not detected in S and G2 phases, all the bands detected with this antibody displayed a stronger signal in mitosis, parallel to the increase of CDK1 activity in mitosis (Fig. 4d). Most of the detected bands (especially bands 5 and 3, but also 4, 7, and 8) respectively revealed a significant drop of intensity in mitotic *Mastl*^*NULL*^ MEFs when compared to WT MEFs (Figs. 4d, f and S5B–E). However, few bands followed a different pattern in mitotic *Mastl*^*NULL*^ MEFs (Fig. 4d, bands 1, 2, and 6, Figs. 4g and S5B, C). The immunoblotting of primary MEFs with this antibody against the CDK phospho-motif further confirmed the reduction of the phosphorylation of different proteins containing this motif (Fig. S5F–H). Furthermore, blotting for phosphorylated S139 in H2AX appeared to be slightly higher in *Mastl*^*NULL*^ cells than in WT cells, independently of the synchonization method (Fig. S5C, lanes 6–9 vs. 1–4).

#### Loss of *Mastl* reduces the activty of NEK2 in early mitosis

**Fig. 5:**
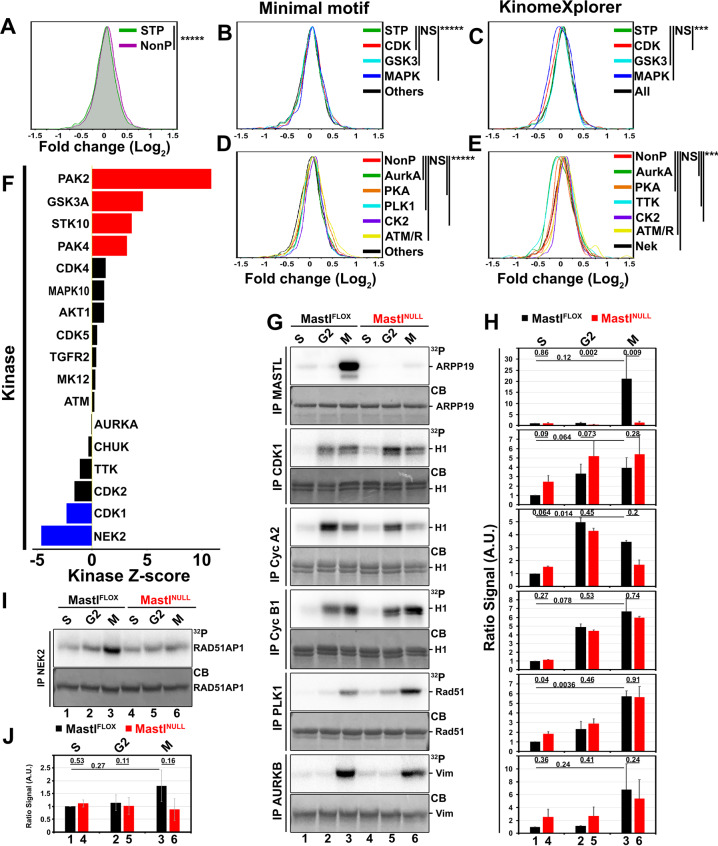
The activity of the mitotic kinome is perturbed by the loss of *Mastl*. Scaled distribution of the phosphosites as a subset of **a** the presence of a proline at position +1, **b**, **c** predicted as targets for various proline-directed kinases or **d**, **e** for selected non-proline-directed kinases using **b**, **d** minimal motifs or **c**, **e** the KinomeXplorer algorithm ***P ≤ 0.0001, ****P ≤ 0.00001, *****P ≤ 0.00001. **f** Bar graph for kinase set enrichment analysis (KSEA) depicting the *Z* score of kinases predicted to present an altered activity based on the fold-change of each phosphosite. **g** Representative kinase assay on the indicated substrate using immunoprecipitated kinase using antibodies directed against the indicated proteins. **h** Bar plot of the relative radioactive intensity of three replicates of the kinase assays from **g** [*N* = 3]. **i** Representative kinase assay for immunoprecipitated NEK2 from stably transduced *Mastl*^*FLOX*^ and *Mastl*^*NULL*^ iMEFs that inducibly expressing a wild-type murine NEK2-Myc. **j** Bar plot of the relative radioactive intensity of three replicates of the kinase assays from **i** [*N* = 3]. ^32^P Radioactive signal. CB Coomassie blue. Error bars depict SEM. pValue obtained from paired *t*-test on log2-transformed values from separate experiments.

MASTL indirectly regulates phosphorylation events essential for mitotic entry and progression as well as cytokinesis by affecting PP2A via ENSA/ARPP19. We previously described how the activity of the MPS1/TTK kinase is altered in the absence of *Mastl*, weakening its action on the spindle assembly checkpoint. This allowed cells to exit mitosis without proper chromosome segregation [21]. Because of the variety of defects observed after the loss of *Mastl*, especially the observation of chromosomal fragments, the decreased activity of MPS1/TTK is unlikely the only source of the phenotype. Therefore, we further analyzed our phosphoproteome data in order to evaluate the activity of the kinome at large. The distribution of the phosphorylation sites over their fold-change depending on the presence of a proline in +1 position of the phosphorylated residue unveiled significant differences (Fig. 5a). This separation displayed a substantial shift of the distribution of S/TP phosphorylation sites toward negative fold-changes (Log_2_). We further deepened the analysis by evaluating the distribution of phosphosites predicted as targets for well-known kinases, clustered as proline-directed kinases or other kinases. The predictions were obtained using either a list of minimal motifs (Fig. 5b, d and Table S2) or the KinomeXplorer algorithm (Fig. 5c, e and Table S2). While the distribution of phosphosites with a CDK motif predicted by the KinomeXplorer was considered significantly shifted to the left in comparison to the entire subset of S/TP phosphosites (Fig. 5c), this was not the case when we compared the distributions using only a more stringent minimal CDK motif (Fig. 5b). The distribution of MAPK minimal motifs was significantly different using minimal motifs, in contrary to when the KinomeXplorer algorithm was used, even though a wider distribution could be observed (Fig. 5b, c). Similar differences were also observed for non-proline-directed kinases (Fig. 5d, e). Only the distribution for CK2 motifs were considered significantly different using both methods. In contrast to the enrichment analysis observed in Fig. 4c, motifs for ATM/ATR kinases were not considered different by applying both methods whereas a shift toward upregulated fold-change values could be observed for both distributions (Fig. 5d, e; yellow line). As a confirmation of our previous report, the distribution for motifs of TTK/MPS1 predicted by the KinomeXplorer algorithm showed a significant difference to the entire subset of non-proline phosphosites (Fig. 5e).

However, such targeted analysis could miss numerous other potential-regulated kinases. Using the KinomeXplorer prediction scores to perform a kinase set enrichment analysis (KSEA, Table S4), we uncovered other kinases that might either be directly regulated or of which the phosphorylation of their substrates would be affected by phosphatases (Fig. 5f). The KSEA revealed PAK2 as the most enriched kinase associated with hyperphosphorylated substrates. Other kinases were also significantly enriched such as GSK3A, STK10, and PAK4. On the other hand, beside CDK1, NEK2 appeared to be the most enriched kinase targeting hypophosphorylated substrates (Fig. 5f). This enrichment analysis also confirmed the reduced activity of TTK/MPS1 in the absence of *Mastl* [21].

The mechanism of action of MASTL assumes that the absence of this kinase would allow a rise of PP2A/B55 activity triggering a reduced phosphorylation status of many substrates. Since kinases themselves are often regulated by phosphorylation events, this could directly or indirectly affect their activity. Therefore, to decipher the causes of the congression defects, we specifically focused on kinases that are connected to hypophosphorylated substrates. Therefore, we determined the activity of MASTL, CDK1, or other mitotic kinases from *Mastl*^*FLOX*^ and *Mastl*^*NULL*^ MEFs collected at three separate cell-cycle phases (for cell-cycle phase enrichment see Fig. S5A). As expected, the activity for MASTL abruptly rose in mitosis in WT cells (Fig. 5g, h) but due to the loss of the protein in *Mastl*^*NULL*^ cells, its activity was barely detectable. Only minimal activity was observed in S and G2 phases. This low activity of MASTL could be in line with recent reports of MASTL activity outside mitosis [25, 30, 39] although this activity was not further reduced in *Mastl*^*NULL*^ MEFs (Fig. 5g, h). CDK1 activity gradually increased from S to G2 phase and peaked in mitosis in WT cells. In *Mastl*^*NULL*^ cells, CDK1 activity was increased by 73%, potentially due to a compensation imposed by the increased PP2A activity. However, such a trend was not observed for the kinase activity associated with cyclin B1. The activity associated to cyclin A2, as previously described [21], was reduced after the loss of *Mastl* even though it peaked in the G2 phase. The activity of PLK1 and AURKB dramatically increased in mitosis but no significant difference was observed between WT and *Mastl*^*NULL*^ cells from the three separate cell-cycle phases (Fig. 5g, h).

Based on our KSEA results, we chose to evaluate the activity of NEK2 more thoroughly. Interestingly, the activity of NEK2 increased in mitosis compared to S phase in WT cells. Surprisingly but confirming our phosphoproteome analysis, NEK2 activity never exceeded background in *Mastl*^*NULL*^ MEFs (Fig. 5i, j), indicating that NEK2 activity is controlled either directly or indirectly by MASTL. Since this is a novel and unexpected finding, we validated the importance of the activity of NEK2 in the development of non-congressed fragments in the absence of *Mastl*. WT MEFs were treated with two different NEK2 inhibitors (rac-CCT 250863 and NCL-0001709 [40, 41]) at two different concentrations after the release of the cells from the DTB (Fig. S6A, B). Counting of cells with non-congressed fragments after NEK2 inhibition revealed a consistent increase in their percentage after exposure of cells to either inhibitor (Fig. S6A, 32–34 vs. 20% in untreated cells). This treatment also modified the distribution of the number of fragments per cell (Fig. S6B). However, both these results were not able to recapitulate the phenotype of *Mastl*^*NULL*^ cells in its entirety. This could be due to several reasons such as the efficacy of the NEK2 inhibitors, the pleiotropic effects of increasing PP2A activity, a premature reactivation of PP1 through its phosphorylation or binding inhibitors or even through ATM/ATR activation. Taken together, our data indicates that the loss of *Mastl* affects mitotic phosphorylations not only through PP2A but also by indirectly affecting the activity of other mitotic kinases, which in turn leads to downstream effects.

#### NEK2 and CDK1 phosphorylate most of the downregulated posphorylation sites in the absence of *Mastl*

**Fig. 6:**
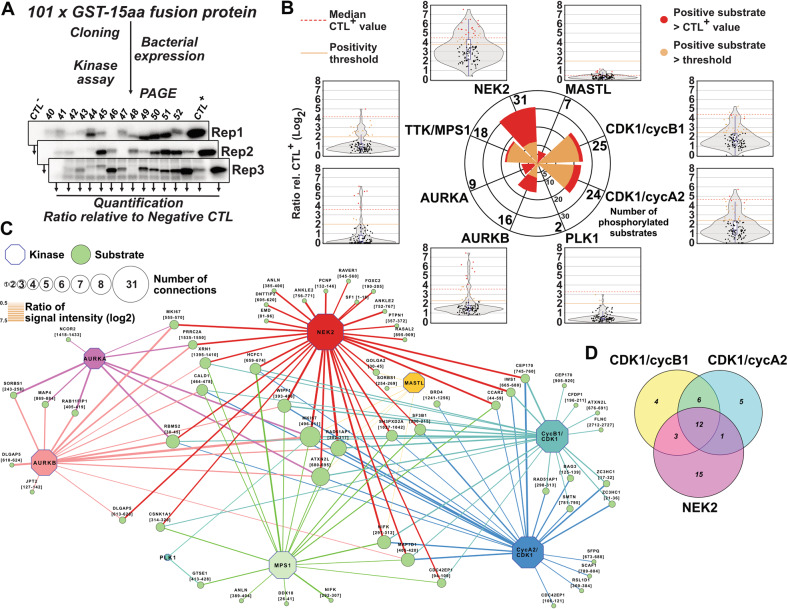
CDK1 and NEK2 target a large panel of phosphorylation sites affected by *Mastl* loss. **a** Cartoon depicting the process in order to test 101 downregulated phosphosites by radioative kinase assays using eight different kinases (see Figs. S7–S9). **b** Polar bar plot of the number of positive substrates for each kinase with their corresponding jitter plot of the mean of the ratio of the intensity for each susbstrate over a violin and box plot depicting their distribution. Red and orange dots indicate positively phosphorylated substrates for the corresponding kinase based on (red) a higher ratio than the positive control and (orange) a ratio value greater than a set threshold (see “Materials and methods”). **c** Kinase-target network connecting 65 positively phosphorylated substrates by a panel of eight kinases. **d** Venn graph of substrates targeted by CDK1/cycB1, CDK1/cycA2, and NEK2.

Although tools that predict potential kinase targeting specific substrates exist, discrepancies remain between literature, empirical data, and in silico predictions. Over the years, only a small number of substrates have been validated for various of the described kinases especially for MASTL, TTK/MPS1, and NEK2. This lack of empirical validation and some conflicting reports makes it difficult to properly assign phosphorylation sites to a specific kinase activity. This generates a vacuum of information, which needs to be filled. Therefore, we decided to evaluate the downregulated phosphorylation sites as substrates of mitotic kinases in order to unveil new associations that could be connected to the observed chromosomal aberrations and congression defects. All the sequences for the unique downregulated phosphorylation sites were extracted (Tables S2 and S5), thoroughly aligned, and compared to the translated sequence of the retrieved correct coding transcript. From these aligned matches, we extracted the sequences coding for 15 amino acid peptides with the phosphorylated residue in their center and subsequently we cloned these fused to the C-terminus of a GST sequence (Fig. 6a and Table S5). These 101 GST-fusion proteins, once expressed and purified, were used as substrates in in vitro kinase assays using radiolabelled ATP, separated on polyacrymalide gels before the quantification of the associated radiointensity by phosphoimaging (Figs. 6a, b, S7–S9, and Table S5). The immunoprecipitated active MASTL kinase phosphorylated its control ARPP19, but showed only background signal intensity for the overall panel of substrates (Figs. 6b and S9A). The difference of signal between the positive and negative controls used for the MASTL assay was minimal, generating only a low ratio in comparison with the other tested kinases (Figs. 6b and S9A). As ENSA and ARPP19 are the only known targets of MASTL, we further evaluated the phosphorylation of six of the most phosphorylated substrates by MASTL (see Table S5). Since the active MASTL was purified by immunoprecipitation from lysates of overexpressing 293T cells arrested in mitosis, we wanted to rule out the possibility that other kinases, especially CDK1 would co-immunoprecipitated with MASTL. To achieve this, we evaluated the phosphorylation of these six substrates in the presence of a WT or kinase dead (KD) version of MASTL. Furthermore, as all these substrates were similarly phosphorylated by CDK1 complexes (Figs. S7 and S8), we extended the assay with WT MASTL in the presence of RO-3306 or GKI-1 to inhibit CDK1 or MASTL, respectively (Fig. S10). Although the six assayed substrates were weakly phosphorylated by WT MASTL, none of them once incubated with KD MASTL displayed a decreased signal markedly higher that the reduction obtained while inhibiting CDK1. This indicates that we were not able to identify new MASTL substrates under the conditions used here.

We therefore evaluated the panel of downregulated phosphorylation sites as targets for other mitotic kinases (Figs. 6b and S7, S8). The pattern of phosphorylation of the panel of substrates by CDK1/cycB1 and CDK1/cycA2 complexes appeared similar, indicating that these kinases have similar substrate specificity in vitro (Figs. S7, S8, and S9A, C). Among the substrates, several previously known targets were confirmed such as MKI67 on S503 and TPX2 on T369 [42, 43]. However, this kinase assay unveiled novel substrates that were phosphorylated by CDK1 complexes such as CEP170 on T752, RAD51AP1 on T305-S309, SF3B1 on T207, and NIFK on S304 (Fig. 6c). Comparison of the in vitro results with the KinomeXplorer prediction score revealed interesting differences (Fig. S9A vs. B). The three most phosphorylated substrates by CDK1 (Figs. 6c and S9A: CEP170, RAP51AP1, and SF3B1) were not predicted as CDK1 substrates by the KinomeXplorer algorithm (Fig. S9B). In contrast, several of the predicted sites by this algorithm were only weakly phosphorylated in vitro such as RACGAP1 on T278 and PTPN1 on S364 (Figs. S7–S9). The divergence between in vitro and in silico results/predictions suggested that further evaluation was warranted. The phosphorylation of the panel of hypophosphorylated substrates was evaluated using PLK1, AURKA, AURKB, TTK/MPS1, and NEK2 (Figs. 6b, S7, S8, and S9A). PLK1 kinase activity was selective and only GTSE1 on T420 and MKI67 on S561 were well phosphorylated by PLK1 (Figs. 6b, c, S7, and S8). GTSE1 is not a surprising target as it was previously reported to be phosphorylated by PLK1 on S435 in the recovery of G2/M cell-cycle arrest induced by p53 [44]. MKI67 is a well-known substrate for mitotic kinases that induces the formation of the perichromosome [45–47]. In this context, it is expected that MKI67 would be phosphorylated by most of the tested kinases with the highest signal attributed to AURKB (Figs. 6c and S9A). Although AURKB showed a relatively high background signal for the entire panel (Fig. 6b), several of the assayed substrates such as DLGAP5, RBMS2, and RAB11FIP1 displayed the most intense signal of phosphorylation (Fig. 6c and S9A). DLGAP5, also known as HURP, was previously reported to be stabilized by AURKA through phosphorylation [48–50]. AURKA displayed a similar pattern of phosphorylation on the panel of substrates when compared to AURKB with a few minor exceptions. SORBS1 on S250 appeared to be the most phosphorylated substrate by AURKA (Figs. 6c and S9A, C) even though no function of SORBS1 in cell proliferation or mitosis has been described. TTK/MPS1 displayed a low intensity of phosphorylation over the panel (Fig. S9A). Nevertheless, 18 targets such as HCFC1 on S666 and NIFK on T299 and S304, were phosphorylated at reasonable level (Figs. 6b, c and S9A). Although none of these specific phosphorylation events by TTK/MPS1 have previously been reported, both HCFC1 and NIFK are known to be regulated during the cell cycle [42, 51–54]. From these two proteins, only NIFK was predicted as a substrate of TTK/MPS1 by the KinomeXplorer (Fig. S9B). NIFK binds MKI67 and relocalizes to the perichromosome during the mitosis similarly to the rRNA to which NIFK associates during interphase [46, 51, 52, 55]. Phosphorylation on S666 of HCFC1 has been abundantly identified over the years by mass spectrometry as reported in PSP database (62x on Jan 2020) and is considered downstream of various signaling including ionizing radiation or nocodazole arrest [56–58]. Its loss causes mitotic defects such as defective chromosome alignment and segregation but how it functions and is regulated remains unknown [53, 54]. Like AURKB, NEK2 kinase displayed a high background signal but 31 of the substrates showed an enriched phosphorylation signal (Figs. 6b, c and S9A). The RAD51AP1 peptide [302–317] was the most phosphorylated NEK2 substrate of the panel but this was not predicted in silico (Fig. S9B and Table S2). Similarly, WIPF1 on S399 was efficiently phosphorylated in vitro but failed to be predicted by the KinomeXplorer whereas both serines of MKI67 were phosphorylated by NEK2 as predicted (Figs. 6c, S8, and S9A, B). When comparing the substrates phosphorylated by CDK1/cyclin B1, CDK1/cyclin A2, or NEK2 (Fig. 6d), only half of the CDK1 and NEK2 substrates overlapped. This indicated unexpectedly that numerous of the phosphorylated substrates by NEK2 contained a proline in +1 position (in 83% of the phosphorylated substrates, Fig. S9D), contrasting with previous studies reporting the absence of such residue in the phopshorylated motif targeted by NEK2 [59, 60].

Our in vitro kinase assays further confirmed some of the in silico predictions from the KinomeXplorer and uncovered novel phosphorylation events, especially for NEK2. Our data revealed that RAD51AP1 is phosphorylated by NEK2 and CDK1 but the impact of its phosphorylation needs to be further studied.

### RAD51AP1 is timely de/phosphorylated in mitosis affecting its binding to RAD51

Identified as a nucleic acid binding protein as well as an interacting partner of the RAD51 recombinase, RAD51AP1 was shown to be an essential binding partner to RAD51 by stimulating its recombinase activity and recruiting other factors [61–66]. Loss of *Rad51ap1* in cells leads to higher cytotoxicity after genotoxic treatment [62, 67] and induces chromosomal breaks in a similar fashion to what we observed in the absence of *Mastl*. Although RAD51AP1 is unlikely the only affected protein of which the regulation could explain the observed defects in *Mastl*^*NULL*^, it is noteworthy that the affected phosphorylation sites T305 and S309 of RAD51AP1 fall within the RAD51 binding motif [293–331 in mouse, 311–349 in human]. Both phosphorylated residues are in close proximity to amino acids such as R315 and L318 (in the mouse sequence) that are crucial for RAD51 binding [62]. T305 of mouse RAD51AP1 is part of a canonical CDK motif (^305^pTPAK^308^) while the motif around S309 differs in the +3 position (^309^pSPSQSL^314^).

**Fig. 7:**
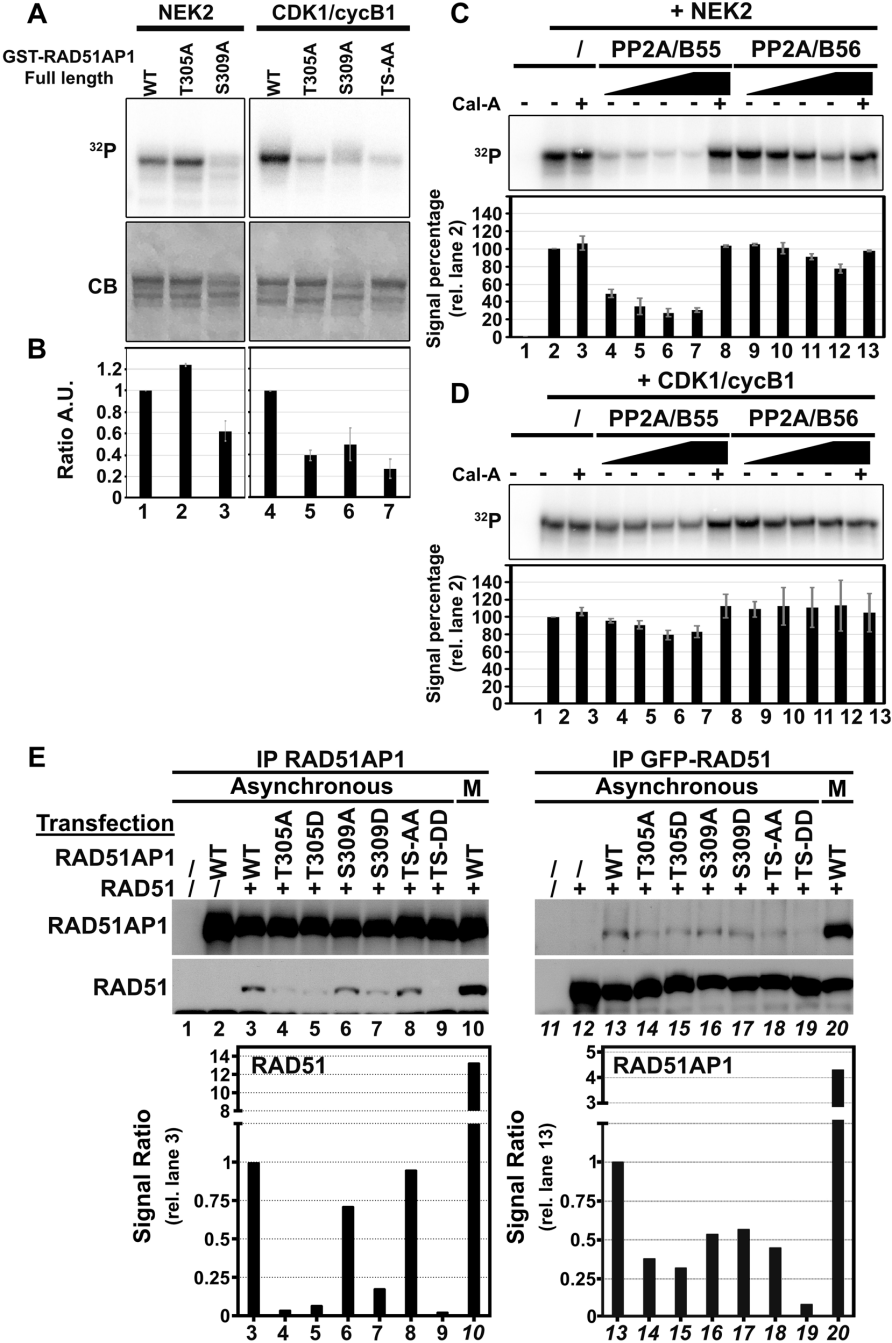
Phosphorylation of RAD51AP1 by NEK2 and CDK1 affects its binding to RAD51. **a** Representative images of kinase assays for wild-type and mutants of full-length murine RAD51AP1 fused to GST and phosphorylated by CDK1/cycB1 complexes or NEK2. ^32^P Radioactive signal. CB Coomassie blue. **b** Bar graph of the averaged ratio of radioactive signal of the mutants vs. the wild-type form from three replicates as shown in **a**. Error bars depict SEM. Phosphatase assay with increasing quantity of immunoprecipitated PP2A/B55 or PP2A/B56 complex in the absence or presence of calyculin A (Cal-A) on prior phosphorylated wild-type full-length murine RAD51AP1 fused to GST phosphorylated by **c** NEK2 or **d** CDK1/cycB1. Error bars depict SEM. **e** Representative blots of co-immunoprecipitation of the RAD51-RAD51AP1 complex transiently expressed in asynchronous or mitotic (M) 293FT cells. Bar graph depicts the signal ratio obtained of the co-immunoprecipitated protein in comparison to the WT asynchronous condition (lane 3).

In light of the correlations of the role of RAD51AP1 in homologous recombination and the observed *MastlKO* phenotype, we further investigated the regulation of phosphorylation in RAD51AP1. The full coding sequences of the WT or mutant murine RAD51AP1 were cloned, GST-tagged, expressed, and purified. The purified proteins were then subjected to a kinase assays using NEK2 or CDK1/cycB1 complexes using radiolabelled ATP as a substrate. While both kinases phosphorylated the WT RAD51AP1, the results obtained using non-phosphorylatable mutants differed (Fig. 7a, b). Whereas T305A was phosphorylated as well as WT by NEK2, the S309A mutant revealed a 40% reduction in signal, indicating that T309 is specifically phosphorylated by NEK2. In contrast, both single T305A and S309A mutants displayed more than 70% signal reduction when using CDK1/cycB1 complexes (Fig. 7a, b). Although the phosphorylation of RAD51AP1 yielded interesting results, we also wanted to study the dephosphorylation of RAD51AP1. Once the phosphorylated WT form of RAD51AP1 was subjected to dephosphorylation, a marked difference appeared depending on which kinase or phosphatase was used (Fig. 7c, d). The WT murine RAD51AP1 was first phosphorylated by NEK2 or CDK1 with the obtained signal intensity serving as a reference (Fig. 7c, d, lane 2). This phosphorylated protein was then further incubated with PP2A/B55 or PP2A/B56 complexes to dephosphorylate RAD51AP1. Since previous studies have reported the action of MASTL through ENSA/ARPP19 on PP2A/B55 complex [8, 9], it was no surprise to observe a lack of activity of the PP2A/B56 complex (Fig. 7c, d, lanes 9–12). In contrast, dephosphorylation of RAD51AP1 by PP2A/B55 was more efficient than with PP2A/B56. RAD51AP1 phosphorylated by NEK2 was efficiently dephosphorylated even with minimal amounts of the PP2A/B55 complex (Fig. 7c, lanes 4–7; Fig. S11A). In contrast, RAD51AP1 phosphorylated by CDK1 was only minorly dephosphorylated by PP2A/B55 (Fig. 7d, lanes 4–7), indicating that PP2A differentiates between phosphates added by NEK2 and CDK1, respectively. Addition of PP2A inhibitor Calyculin A (Fig. 7c, d, lanes 8 and 13) inhibited the observed effect, suggesting that PP2A activity is required for this process.

To further decipher the role of T305 and S309 phosphorylation and their potential effect on the binding of RAD51AP1 to RAD51, we co-expressed GFP-tagged RAD51 with WT or mutant forms of RAD51AP1 fused to mCherry in HEK293T cells (Fig. S11B). Co-immunoprecipitation of the RAD51-RAD51AP1 complex revealed differences in binding between the partners depending on the mutant in asynchronous cells (Fig. 7e). When compared to the WT form, most of the assayed mutants displayed reduced binding between RAD51AP1 and RAD51. As expected, an obvious increase of the binding partner RAD51, can be observed once co-expressed (Fig. 7e, lane 3 vs. 2 and lane 13 vs. 12). The single non-phosphorylatable mutant T305A reduced the binding of RAD51 to RAD51AP1 by 90% (lane 4) and the binding of RAD51AP1 to RAD51 by 62% (lane 14). Unexpectedly, the phosphomimetic mutant T305D displayed a similar reduction of interaction with RAD51 (lanes 5 and 15). Immunoprecipitation of the RAD51-RAD51AP1 complex with S309A or S309D mutants (lanes 6–7 and 16–17) revealed a reduced interaction of RAD51 but differed in their strength. However, once both sites were mutated to become phosphomimetic residues (T305D/S309D, lanes 9 and 19), almost no interaction between RAD51AP1 and RAD51 can be observed. The interaction of RAD51 and RAD51AP1 appeared to be substantially stronger when the co-transfected cells were arrested in mitosis with nocodazole (Fig. 7e, lanes 10 and 20). During mitosis, the intensity of the interacting proteins increased between 4 and 13 times depending of the immunoprecipitated protein in comparison to the same interaction in asynchronous cells (Fig. 7e, lane 10 vs. 3 and lane 20 vs. 13), suggesting that the RAD51/RAD51AP1 complex may have important functions in mitosis.

## Discussion

Our study reveals a global picture of the importance of the Greatwall kinase/Mastl balancing phosphorylation events during mitosis. Its loss results in the formation of micronuclei that give rise to multilobulated, multinucleated cells and eventually leading to cell death. Cells lacking *Mastl* were also reported to form chromosome bridges with lagging, mis-aligned, and decondensed chromosomes during mitosis [18, 21, 22, 68]. Our analysis of the chromosomes of *Mastl*^*NULL*^ MEFs uncovered the appearance of fragments. These were deprived of centromere staining, unattached to the microtubule spindle, and lying outside the metaphase plate or the monoastral ring of congressed chromosomes and therefore are most likely the source of the mirconuclei. The lack of a proper kinetochore on these fragments likely explains that they are unattached to microtubules, therefore disabling their congression to the metaphase plate and their proper segregation. Such acentric chromosomes/fragments mainly result from chromosome breaks due to irradiation or replication stress that are improperly repaired before the mitosis [69]. Enrichment term analysis of our phosphoproteome data indicated that most of the affected phosphorylated proteins were involved in mitosis and cell cycle. However, a smaller cluster linked to double-strand break DNA repair, was also enriched with hypophosphorylation of RAD51AP1 and SFPQ. This suggests that the activation of the DNA damage repair process may contribute to the *MastlKO* phenotype. In parallel, a significant number of ATM/ATR targets were upregulated in the absence of *Mastl*, further confirming involvement of the DNA damage response. However, the observed upregulation of phosphorylated SQ sites contrasts with the model of action of MASTL inhibiting PP2A/B55 through ENSA and ARPP19 and therefore we do not understand yet how ATM/ATR contributes to this. In addition, our results confirm a significant number of downregulated TP phosphorylation sites that are specifically targeted by PP2A/B55 in mitosis [32, 34, 70].

Although multiple proteins with downregulated phosphorylation were targeted by CDK1, others were predicted and phosphoryated in vitro by TTK/MPS1 and NEK2. Similarly to our previous study reporting the decreased activity of TTK/MPS1 due to a lack of phosphorylation that weakens the SAC [21], the activity of NEK2 also appeared to be reduced in the absence of *Mastl*. Both MPS1/TTK and NEK2 participate in the alignement and segregation of chromosomes [71–74]. Nevertheless, NEK2 is better known for its action on the disjunction of centrosome by phosphorylating CEP250/c-Nap1 and Rootletin/CROCC at the onset of mitosis [75, 76]. Our results suggest broader functions of NEK2 on multipe novel targets in addition to the 13 proteins currently referenced in the PhosphoSitePlus database. Among the 101 tested substrates, 23 of them displayed a more intense signal than the positive control. However, 19 of the 23 sites phosphorylated by NEK2 constrast with previous studies reporting the necessity of the absence of a proline at +1 [59, 60], as all 19 peptides contained a proline after the phosphorylated residue. Other phosphorylation sites with a proline at +1 of the phosphorylated residue have also previously been referenced on PhosphoSitePlus as targets of NEK2 including DVL3 on T15, P53 on S315, SGO1 on S507 [71, 77, 78]. Among the novel substrates of NEK2 in our study, RAD51AP1 was most efficiently phosphorylated by NEK2, especially on S309, with T305 and S309 presenting a proline after the phosphorylated residue. RAD51AP1 was not only phosphorylated in vitro by NEK2 but also by CDK1 on both residues. Phosphorylation on T305 and S309 of RAD51AP1 was primarily detected in mitosis in our study as well as in others and appears unstable during mitotic exit or after PLK1 inhibition [32, 56, 57, 79].

The periodicity of CDK1 activity, peaking in mitosis, likely explains the T305/T309 phosphorylation in RAD51AP1. On the other hand, the periodicity of NEK2 activity contrasts with the mitotic phosphorylation of RAD51AP1. NEK2 has been shown to display a periodic activity through the cell cycle; peaking in S phase, reduced in G2, and increasing again in early mitosis [80–82]. Intriguingly, our results differ from this observation as NEK2 activity appeared to peak in prometaphase-arrested cells. Like TTK/MPS1, NEK2 is phosphorylated on multiple sites either by PLK1 or by autophosphorylation [81, 83, 84] and is dephosphorylated by PP1, reducing its activity [85, 86]. Under normal conditions, PP1 remains inactive during mitosis due to its inhibiting phosphoylation on T320 (PPP1CA) by CDK1/cycB1 [87, 88] and only upon the drop of cyclin B1 expression, PP1 is able to reverse the balance, by auto-dephosphorylating itself. However, no change in phosphorylation on T320 of PP1 was observed from our phospho-proteomic analysis. In parallel, our data indicated the modulation of phosphorylation of three regulators of PP1, namely, SDS22 (Ppp1r7), IASPP (Ppp1r13l), and IPP2 (Ppp1r2). Both SDS22 and IPP2 have been reported to be regulated after ionizing radiation, leading to their phosphorylation by ATM and dissociation from PP1 [89, 90]. However, only SDS22 displayed a significantly increased phosphorylation on S12, a predicted ATM site. From our results, we can only speculate that the observed marginal increase in ATM activity would sufficiently activate PP1 to explain the reduced activity of NEK2 in the absence of *Mastl*.

Among the affected phosphoproteins associated with double-strand DNA damage repair in the absence of *Mastl*, SFPQ and RAD51AP1 are both regulators of homologous recombination that bind to RAD51 [63, 91, 92]. However, only the phosphorylation of RAD51AP1 was regulated in its RAD51 binding motif. RAD51AP1 is an essential binding regulator of RAD51 that enhances its homologous recombination activity [62, 63] by binding directly the DNA and recruiting other partners [61, 65, 93]. Deficiency of RAD51AP1 in cells impairs homologous recombination and leads to chromosome breaks and fragments visible in mitosis as it was observed in the absence of *Mastl* [62]. Despite its action, the role of RAD51AP1 phosphorylation during mitosis is unclear. Our results indicate that the interaction between RAD51 and RAD51AP1 was stronger in mitosis. Our data only allow us to speculate that RAD51AP1 phosphorylation could also affect the DNA binding capablity of RAD51AP1, allowing RAD51 to dissociate from DNA during mitosis. In the absence of *Mastl*, this would lead to a persistent attachement of the RAD51/RAD51AP1 complex onto chromatin, potentially leading to aberrant stochastic recombination events.

In summary, our data link multiple molecular processes that are affected by the absence of *Greatwall kinase/Mastl*. The lack of this kinase creates a disequilibrium in the overall phosphoproteome by affecting the activity of the PP2A/B55 as well as of other kinases in mitosis. The unbalance caused by *Mastl* deficiency is exacerbated over time, leading to defects that are first visible in mitosis and continue to accumulate, making it impossible to repair them at the later phase of the cell cycle. The emerging idea to inhibit *Mastl* as an anticancer target has recently shown appealing results to reduce tumor size and tumor invasion in various models [22, 24, 25]. However, because of its broad effects in mitosis, targeting *Mastl* might also affect normal cells inducing detrimental effects in patients. Aside the current lack of specificity and similarity of effects of the first generation of MASTL inhibitors, its combination with genotoxic treatment, the titration of the drug in parallel to the evaluation of the degree of inhibition of MASTL, especially in breast cancer overexpressing MASTL, will need to be investigated in the future [19, 21].

## Materials and methods

### Cell culture

Primary mouse embryonic fibroblasts (pMEFs) of the *Mastl*^*FLOX/FLOX*^ *Esr1-CreERT2* genotype were isolated as previously described [2, 21]. Primary *Mastl*^*FLOX/FLOX*^ MEFs were immortalized (iMEFs) by serial passaging for 30 times using a modified 3T3 protocol as previously described [21, 94]. pMEFs, iMEFs, and 293T cells were cultured in DMEM (HyClone SH30243.01) supplemented with 10% fetal bovine serum (Gibco 26140–079) and 1% penicillin/streptomycin (Nacalai Tesque 09367-34). All cells were cultured under humidified atmosphere with 5% CO_2_ and 3% O_2_ for pMEFs or 21% O_2_ for all other cells.

### Cell-cycle synchronization

Primary and immortalized MEFs were synchronized at the G0/G1 phase of the cell cycle by culturing at high confluence (contact inhibition) and starvation in reduced serum containing growth media (0.2% fetal bovine serum) for 72 h. In paired experiments comprising *Mastl*^*FLOX/FLOX*^ and *Mastl*^*NULL/NULL*^, identical primary MEF clones or immortalized MEFs were treated with ethanol or 100-ng/ml 4-OHT (Sigma, H7904) to obtain *Mastl*^*FLOX*^ and *Mastl*^*NULL*^ cells, respectively. To induce synchronized entry into cell cycle, cells were trypsinized and replated at a lower cellular density in the presence of 10% FBS. Mitotic arrest was achieved by the addition of mitotic poisons (500-ng/ml Nocodazole [Sigma M1404], 5-µM Eg5 inhibitor II/STLC [Calbiochem 324621], or 100-ng/ml Demecolcine [Sigma D1925] between 20 and 24 or 24 and 28 h after release for primary or immortalized MEFs, respectively, and the cells were subsequently collected and processed.

Synchronization of cells in S phase was performed by DTB. Cells were seeded 24 h before the first thymidine block and then treated with 4-mM thymidine for 18 h. Cells were released by two washes (1x with PBS, 1x with DMEM) and incubated in 10% FBS DMEM for 10 h. Deletion of *Mastl* (*Mastl*^*NULL*^) was achieved by addition of 100-ng/ml 4-OHT during the first release. Cells were blocked again for 16 h by addition of 4-mM thymidine. Released cells (1 wash with PBS, 1 wash with DMEM) from the second block were arrested in mitosis by addition of mitotic poisons 8-h post release from the second block for 4 h and then collected.

Synchronization at the G2/M transition was achieved by releasing cells from the DTB second block in presence of 9 µM of RO-3306 for 12 h. Cells were then released from this block (one wash with PBS, one wash with DMEM) to be collected either in mitosis in presence or not of mitotic poissons after 45 min or in the next cell cycle after 16 h.

### Lentiviruses production, transduction, and generation of stable cell lines

Tamoxifen (4-OHT)-inducible *Mastl* knockout iMEFs were obtained as previously described [21] after retroviral infection (pWZL-CreERT2 [PKB931]) and blasticidine selection (10 µg/ml, Invivogen). For lentiviral transduction of iMEFs, 293T cells were transiently transfected for 8 h by the calcium phosphate method of a mix of three plasmids (psPAX2 [PKB2019], pMD2G [PKB1598], and a plasmid coding for the gene of interest [see Table S6]). Lentiviruses were harvested 48 h after transfection, the collected supernatants were ultracentrifuged (23,000 rpm, 2 h, 4 °C) and pellets were resuspended in 10% FBS-containing DMEM before infection of 500 × 10^3^ cells.

### Transient transfection

One day after seeding, 293T cells were transfected by calcium phosphate method. For protein intended for kinase assay (Mastl WT and KD, PLK1), 30 µg of plasmid were used per transfection of 2.5 × 10^6^ cells in 15-cm dish. For protein intended for phosphatase assay (PP2A-A, PP2A-C together with B55 or B56), a total of 36 µg of plasmids was transfected (see Table S6 for proportion). Sixteen hours before lysis cells were arrested in mitosis with 500 ng/ml of nocodazole. For expression of active PP2A complex, transfected 293T cells were further treated with 7-µM RO-3306 for 20 mins before lysis to induce premature mitotic exit and obtain full activity of PP2A complex.

### Immunofluorescence microscopy

For interphase cells, iMEFs were seeded in 3-cm dish (Nunc) and synchronized by DTB. After the release (DTB 0 h; S phase), 12 h after release in presence of 9 µM of RO-3306 (RO 12 h, G2 phase) or 24 h after release (next cycle), cells were either directly fixed with 10% neutral-buffered formalin (NBF, Sigma HT501128) or subject to pre-extraction for 5 min at room temperature with PHEM (60-mM PIPES, 25-mM HEPES, 10-mM EGTA, 4-mM MgSO_4_, pH 7.0 with KOH) + 0.1% Trion X-100 before fixation in order to lower cytosolic soluble proteins amount and decrease background. Mitotic cells were collected by mitotic shake-off (gentle pipeting up and down for iMEFs), spun and resuspended in PBS before to be directly cytospun on polysine slides (Thermo Scientific) for 2 min at 500 rpm. Cells were either directly fixed or subject to pre-extraction (PHEM + 0.1% Trion X-100) before fixation.

All fixed cells were permeabilized for 3 min at room temperature with 0.5% Triton X-100 in PBS (PBST) and further blocked with PBST-BSA [PBS containing 0.2% Triton X-100, 2% BSA (Sigma A7906), and 10% normal goat serum (JacksonImmunoResearch 005-000-121)]. Primary antibodies (see Table S6) were diluted in PBST-BSA and incubated overnight at 4 °C. Cells were washed three times with PBST for 5 min and incubated with secondary antibody (see Table S5) for 2 h in the dark at room temperature. Cells were counterstained with 2 mg/ml of Hoechst 33342 (Invitrogen H3570) in PBS for 5 min at room temperature, and a coverslip was mounted with Immu-Mount (Thermo Scientific 9990402). Images were acquired either using an Epifluorescence Zeiss Axioimager Z1 microscope equipped with Zeiss AxioCam MRc5 and a 100 × objective (Zeiss, Plan-APOCHROMAT, 100 × /1.4 Oil DIC, ∝/0.17), or with an upright confocal LSM800 microscope. Acquired CZI images were then processed using ImageJ.

### Chromosome spreads

Demecolcine-treated mitotic cells were harvested by mitotic shake-off (gentle pipeting up and down for iMEFs) and spun. Cell pellets were resuspended in warmed 75-mM KCl hypotonic buffer and incubated for 15 min at 37 °C. For immunofluorescence staining, cells were then cytospun at 2000 rpm for 5 min on polysine slides and fixed immediately for 3 min with 10% NBF. For GIEMSA or DAPI staining, 1/10 volume of 3:1 methanol/acetic acid was added to cells followed by centrifugation at 1000 rpm for 15 min. Cells were then fixed by resuspension in 3:1 methanol/acetic acid solution, incubated for 30 min at room temperature, centrifuged at 1200 rpm for 5 min and finally washed once more with fixative. Cells were resuspended in a small volume of fixative, dropped onto clean glass slides and left to air dry. Automated acquisition of chromosome spreads stained with GIEMSA was performed using Metafer imaging platform (MetaSystem).

### Plasmid construction and site mutagenesis

The full-length mouse *Rad51ap1* (NM_009013) coding sequence (CDS) was obtained by PCR amplification from a cDNA preparation from iMEFs. CDS were introduced into a pENTR/D-TOPO^®^ entry vector (Thermo Fisher; k240020) for further mutagenesis and gateway cloned in pBobi vector with C-terminal mCherry tags. The murine *Rad51* CDS was cloned similarly with a EGFP tag in C-terminal.

To test the kinase activity of NEK2, full-length mouse *Nek2* (NM_010892.3) coding sequence was obtained by PCR and cloned similarly than *Rad51ap1*. The *Nek2* coding sequence was futher gateway subcloned in a modified TetOn inducible pLVX-tight-puro vector with a C-terminal Myc tagged. To express NEK2, *Mastl*^*FLOX*^ MEFs were transduced with a rtTA transactivator vector (pLVX-TetOn-Neo) and the pLVX-Nek2-Myc vector and selected with their respective antibiotics resistance. The expression was obtained by the addition of doxycycline from the start of the experiment.

All 15 amino acid peptides derived from mass spectrometry data were inserted in the carboxy-terminal part of the GST coding sequence by ligating annealed complementary oligomers in pGEX-6-P1. Full-length *Rad51ap1* WT and mutants were also introduced in this same vector. Substitution mutations were introduced into plasmid DNA by PCR-based site-directed mutagenesis using the QuikChange Lightning Site-Directed Mutagenesis Kit (Agilent; 210519). A list of all primers and clones can be found in Tables S5 and S6.

### Recombinant protein expression in bacteria

All recombinant protein expression was performed in Dh5α *E.Coli* cells (Invitrogen;18265017). After cell growth to an OD600 of 0.8 in LB broth, 0.5 mM of IPTG was added to induce protein expression for 18 h at 20 °C. Cells were then harvested and lysed in LKB buffer (25-mM Tris-HCl pH 7.5, 100-mM NaCl, 1-mM EGTA, 1-mM EDTA, 0.5% NP40, 25-mM ß-glycerophosphate, 2-mM DTT, 10-µg/ml protease inhibitors [Leupeptin {E18}, Chymostatin {E16}, Pepstatin {E110} from Millipore]), sonicated (5 min with 30 s pulse and 30 s rest at an amplitude of 30%, Branson), and clarified by centrifugation at 15,000 *g* for 30 min at 4 °C. Proteins were purified by incubating the supernatant with glutathione- Sepharose 4B (GE Healthcare; 17-0756-05) for 90 min at 4 °C and washed thrice with LKB buffer without protease inhibitors. GST-fusion proteins were eluted by competition with 10-mM reduced L-glutathione pH 8.0 (Sigma; G4251) added to LKB buffer. All GST-fusion proteins with 15 amino acid peptide were quantified with a filter paper dye-binding assay [95] and diluted at 250 ng/µl with EB buffer (80-mM glycerophosphate pH 7.3, 20-mM EGTA, 15-mM MgCl_2_, 10-µg/ml protease inhibitors (Leupeptin, Chymostatin, Pepstatin)) with 10% glycerol and 1-mM DTT. RAD51AP1 proteins were further diluted in buffer K (20-mM Tris-HCl pH 7.5, 0,5-mM EDTA, 1-mM DTT, 10% glycerol, 0.01% Igepal 100-mM KCl) and then concentrated with an Amicon Ultra-15 centrifugal filter unit (Millipore).

### Recombinant protein expression in insect cells using baculoviruses

Coding sequence for human *Cdk1* (NM_001786), *cyclin B1* (NM_031966), and *cyclin A2* (NM_001237) was introduced in pFASTBac-HT B (Invitrogen; 10584027) as a fusion protein cycB1-CDK1 or cycA2-CDK1. A linker between the cyclin and the CDK1 was added to encode for the amino acid sequence ASKGGGGSLEVLFQGPSR. DH10Bac competent bacteria were transformed with 200 ng of obtained pFastBac-HT-B-cycB1-CDK1 (PKB 1982) and pFastBac-HT-B-cycA2-CDK1 (PKB 1981) and selected on LB agar plates containing 10-µg/ml tetracycline, 50-µg/ml kanamycin, 7-µg/ml gentamicin, and spread with 2.5-mg/ml IPTG and 10-mg/ml X-Gal. Incorporation of the fusion sequence was confirmed in white picked colonies by colony PCR using the primers PKO 4237 and 4238. Bacmid DNA was obtained from verified clones and purified by isopropanol precipitation and transfected in Sf9 insect cells (Gibco, 11496015). Sf9 insect cells were cultured using Sf900 III SFM (Life Technologies; 12658) supplemented with 10% FBS, 0,1% Pluronic F-68, 50-µg/ml gentamicin, and 1% penicillin/streptomycin. 8 × 10^5^ Sf9 cells were transfected with a mix of 2 µg of bacmid and 8-µl Cellfectin II (Gibco; 10362100) in unsupplemented SF900 medium and incubated for 72 h at 27 °C. Baculoviruses stock was then harvested (P0) and amplified three times (P3). For protein expression, 10^6^ Sf9 cells were infected with P3 baculoviruses and incubated for 72 h in the dark under constant stirring. Cells were then harvested, spun, washed in cold PBS twice, and resuspended in cold lysis buffer (500-mM KCl, 20-mM Imidazole pH 8, 20-mM Tris pH 7.4, 20% glycerol, 0.1% NP40, 10-µg/ml protease inhibitors [Leupeptin, Chymostatin, Pepstatin]). Cells were further sonicated (pulse 30 s ON, 1.5 min OFF, amplitude 20%) and centrifuged at 16,000 *g* for 30 min at 4 °C. The supernatant was incubated with washed Ni-NTA agarose beads (Thermo Fisher/Invitrogen R90110) for His-tag pulldown overnight at 4 °C under constant rotation. After washing the beads thrice with cold lysis buffer and once with cold wash buffer (20-mM Tris pH 7.4, 100-mM KCl, 20-mM Imidazole, 20% glycerol, 0.1% NP40, and 10-µg/ml protease inhibitors), fusion proteins were eluted from the beads by incubating 2 h at 4 °C with elution buffer (20-mM Tris pH 7.4, 100-mM KCl, 500-mM Imidazole, 20% glycerol, 0.1% NP40, and 10-µg/ml protease inhibitors). The collected supernatant was concentrated with an Amicon Ultra-15 centrifugal filter unit.

### Immunoprecipitation

Cells were lysed with EBN buffer (EB buffer supplemented with 0.5% NP40 and 150-mM NaCl) with 10% glycerol, 1-mM DTT, 4-mM NaF, and 10-µg/ml protease inhibitors (Leupeptin, Chymostatin, Pepstatin). Lysates were quantified as previously described [95]. Twelve microliter of protein A-agarose beads (Invitrogen 15918-014) were washed and incubated overnight with 0.5–3 µg of primary antibodies (see Table S5) diluted in EBN buffer at 4 °C under constant rotation. Beads were subsequently washed thrice in 1-ml EBN buffer and 150 µg to 1 mg of whole cell lysate were added to the beads for immunoprecipitation during 5 h at 4 °C under constant rotation. After incubation beads were washed with EBN buffer for WB or washed with EB buffer for kinase assay. Finally, “dry” protein A beads were resuspended in 100 µl 1× Laemmli SDS sample buffer and further denaturated at 95 °C for 5 min.

### Kinase assay

Kinase assays with recombinant proteins were performed in EB buffer (containing 15mM Mg^2+^) by incubating 1.25 µg of GST purified recombinant proteins with 35–100 ng of active recombinant kinases (CDK1/cycB1 [homemade or Carna Biosciences 04-102], CDK1/cycA2 [homemade], AURKA/TPX2 [Carna Biosciences 05-186], AURKB/Incenp [Carna Biosciences 05-102], TTK/MPS1 [Carna Biosciences 05-169], NEK2 [Carna Biosciences 05-226]) or with immunoprecipitated MASTL (WT or KD) or WT PLK1. Incubation was carried at room temperature (23 °C) under constant shaking (1200 rpm) for 30 min in presence of 50-µM ATP (Sigma; A2609) and 2 µCi of [gamma-^32^P] ATP (PerkinElmer; BLUE502A). The positive controls for each kinase were GST-ARPP19 [60-75] (2391) for MASTL, GST-Lamin A [12–26] (PKB2302) for CDK1/cycB1 and CDK1/cycA2, GST-RAD51 [7–21] (PKB2338) for PLK1, GST-Vimentin [66–80] (PKB2335) for AURKB, GST-LATS2 [76–91] (PKB2333) for AURKA, GST-TTK [669–684] (PKB2569) for TTK, and GST-NDC80[158–173] (PKB2565) for NEK2.

For kinase assay using endogenous immune-precipitated kinases, agarose beads were resuspended and incubated in EB buffer containing a substrate {histone H1 for CDK1, cycB1, and cycA2 IP; GST-RAD51 [7–21] (PKB2338) for PLK1 IP, GST-ARPP19 [25–112] (PKB1506) for MASTL IP, GST-Vimentin [66–80] (PKB2335) for AURKB IP, and GST-RAD51AP1[302–317] (PKB2487) for NEK2}, 25-µM ATP, and 5 µCi of [gamma-^32^P] ATP. Incubation was carried at room temperature (23 °C) under constant shaking (1200 rpm) for 30 min. Reactions were stopped by addition of 4× SDS Laemmli sample buffer and proteins were separated on 12% SDS-PAGE, gels were dried, and levels of incorporated radioactivity were quantified using a PhosphoImager (Fujifilm; FLA-7000).

### Selection of positively phosphorylated substrates from each kinase assay

To select substrates among the 101 tested GST-fusion peptides, two separate criteria were applied. Substrates with an averaged intensity ratio (signal/negative control) higher or equal than the ratio intensity of the respective positive control were considered positively targeted by the kinase. As such assays create by nature a positively skewed distribution and that a highly phosphorylated positive control would remove less intense phosphorylated substrates, another criteria were applied to determine more modestly phosphorylated substates. The positively skewed distribution was progressively trimmed of the highest value in order to tend to a normal distribution. Following each trim, a skewness score was calculated [skewness, type 3 from e1071 R package (v1.7-3)]. The trim was stopped once the skewness score fell below 1 tending to a normal distribution of the rest of the substrates panel. A threshold was then defined as the percentile 90% of this “normal distribution.” In the case that the obtained threshold value appeared below four times the signal of the negative control, this value would be discarded and exchanged to a threshold ratio value of 4. Positive substrates were defined then when their averaged ratio value was greater or equal to this threshold value for each kinase.

### PP2A phosphatase assay

PP2A phosphatase assays were performed as previously described [21] with minor modifications using an entire immunoprecipitated ectopic PP2A complex with either B55 or B56 as regulatory subunits. Sequence for catalytic and scaffold subunits was obtained from addgene as a gift from William Hahn (pBABE zeo PPP2CA WT, Addgene plasmid 10689 and pMIG-Aalpha WT, Addgene plasmid 10884) [96, 97].

### Immunoblot

Cells were lysed either in Laemmli 1× sample buffer or EBN buffer (80-mM β-glycerophosphate pH 7.3, 20-mM EGTA, 15-mM MgCl_2_, 150-mM NaCl, 0.5% NP40, 1-mM DTT, and protease inhibitors [20 μg/ml each of leupeptin, chymostatin, and pepstatin (Chemicon, EI8, EI6, and EI10)]) and homogenized with 15 strokes using a glass douncer before snap freezing cell lysates in liquid nitrogen and stored at −80 °C. Lysates were centrifuged for 10 min at 12,000 *g* at 4 °C. Ten microgram of protein extract was separated on polyacrylamide gels, transferred onto polyvinylidene difluoride membranes (Millipore, IPVH0010) using a semi-dry system, and blocked in tris-buffered saline with 0.1% Tween20 and 4% nonfat dry milk (Bio-Rad, 1706404). Blots were probed with the appropriate primary antibodies overnight at 4 °C, followed by secondary goat anti-mouse (Pierce, 0031432) or anti-rabbit antibodies (Pierce, 0031462) conjugated to horseradish peroxidase and developed using enhanced chemiluminescence (PerkinElmer, NEL105001EA).

### Mass spectrometry-based quantitative phospho-proteomic analysis using isobaric TMT

#### Mitotic cell collection and protein extraction

Immortalized *Mastl*^*FLOX/FLOX*^ *CreERT2* MEFs were synchronized by DTB as described above and released in 10% FBS DMEM for 4v h before addition of 500 ng/ml of nocodazole for 5 h. Mitotic cells were isolated by pipetting and counted. 10^7^ cells for both *Mastl*^*FLOX*^ (CTL) and *Mastl*^*NULL*^ (KO) were pelleted and snap-frozen in liquid nitrogen for MS-based (phospho)proteomic analysis. A total of three separate experiments with paired samples (*Mastl*^*FLOX*^ vs. *Mastl*^*NULL*^) were performed yielding three biological replicates (BR1, BR2, and BR3). All collected samples (six in total) were processed and analyzed together using a TMT 6-plex scheme (see Fig. 2a).

#### Protein extraction and preparation of peptide samples

Each sample of snap-frozen collected cells was lysed in 1 ml of urea lysis buffer (8-M urea dissolved in Tris-HCl 50-mM pH 8.0). Protein extracts in 8-M urea lysis buffer were sonicated on ice for 15 cycles of 3-s ON, 3-s OFF at an amplitude of 30% (Sonics Vibra-Cell VC750) reduced by addition of TCEP (final 20 mM) for 20 min at room temperature and then alkylated with 55-mM chloroacetamide (RT, in the dark for 30 min). The samples were diluted eightfold to a final concentration of 1 M urea using 100-mM triethylammonium bicarbonate (TEAB pH 8.5; Sigma-Aldrich T7408) before predigestion overnight at 25 °C with 75 µg per sample of Lysyl endopeptidase (LysC, Wako 129-02541). The samples were further diluted at 0.8-M urea by addition of 2-ml 100-mM TEAB, and trypsin digested overnight at 25 °C using 75 µg per sample of sequencing-grade trypsin (Promega, V5117). Before continuing samples prepation, sufficient digestion (<10% miscleavage) were assessed using 50 µl of the 10-ml total sample volume of tryptic digested peptides. Each peptide solution was then brought down at pH 2.0 by addition of trifluoroacetic acid (TFA Sigma-Aldrich T6508; final proportion 1% v/v), desalted using Sep-Pak C18 Cartridge (Waters, WAT051910), and eluted with 4 ml of 80% acetonitril (v/v) and 0.5% acetic acid (v/v).

In order to perform the proteomic analyses, an aliquot of 100 µl of eluted peptides solution (4 ml of eluted volume; 2.5% of total volume) was immediately transferred after elution. All samples were snap-frozen in liquid nitrogen before lyophilization overnight at −80 °C (Christ Alpha 2–4 LDplus).

### Proteomic analysis

#### Isobaric TMT labeling and fractionation

Lyophilized samples obtained from transferred 100 µl of the eluted peptide solution were resuspended in 100 µl of TEAB, and 20 µl of such peptide solution were labeled using a TMT 6-plex isobaric label reagent (Thermo Scientific, 90406). Each samples were labeled with 185 µg of TMTs according to the scheme presented in Fig. 2a and incubated overnight at room temperature before to be quenched by addition of 1-M Tris-HCL pH 7.5.

The labeled samples were combined, acidified, and desalted on Sep-Pak C18 cartridges (Waters, WAT051910). The desalted samples were dried, and resuspended in 5% acetonitrile, 5% ammonium hydroxide solution (pH 10.0), and then pre-fractionated into 80 fractions using a high pH reverse-phase Zorbax 300 Extend-C18 column (5 um, 4.6 × 250 mm, Agilent) on an ÄKTAmicro system (GE Healthcare). The pre-fractionated samples were concatenated into 20 injection fractions for each sample.

#### MS analysis

The 20 concatenated fractions of the combined samples were injected on an Orbitrap Fusion (Thermo Fisher). Each injection was separated on a 50 cm by 75 µm EASY-Spray RP-C18 LC column (Thermo Scientific) in a 75-min gradient of solvent A (0.1% formic acid in water) and solvent B (99.9% acetonitrile, 0.1% formic acid in water) on Easy LC 1000 (Thermo Fisher Scientific), coupled with Obritrap Fusion mass spetrometer (Thermo Fisher Scientific). Survey full scan MS spectra (*m/z* 400–1600) were acquired with a resolution of *r* = 60,000 (at *m/z* 200 Th), an AGC target of 4e5, and a maximum injection time of 100 ms. MS/MS acquisition was acquired in Orbitrap analyzer (AGC target 5e4; HCD 38; *r* = 15,000). A dynamic exclusion was applied exclusion duration of 60 s.

#### MS data analysis

Peak lists were generated using the Thermo Proteome Discoverer software (version 2.1.0, Thermo Fisher Scientific). Spectra were searched using Mascot against target-decoy Mouse Uniprot database. Carbamidomethyl cysteine and TMT 6-plex labeling on N-terminus peptide and lysine were set as fixed modifications. Oxidation (M) and deamidation (NQ) were set as variable modifications. For peptide assignment, minimal length of six amino acids and maximum three missed cleavages were required, while allowing for maximum 30-ppm mass deviation for MS survey scan and 0.06-Da mass deviation for MS/MS ion fragments, respectively. FDR control was performed on both PSM and peptide level at the level of 0.01 for high and 0.05 for medium confidence peptides. The co-isolation threshold for reporter ion quantification was set at 50%. Protein groups were assembled for downstream data analysis (see Table S1).

### Phospho-proteomic analysis

#### Phospho-peptide enrichment

Digested tryptic peptides previously lyophilized were reconstituted in 5-ml 50% acetonitrile, 1.5% TFA, and added to 2 mg of “Titansphere TiO_2_ 5 μm” (GL Sciences Inc., Japan). The mixture was incubated on a rotating wheel for 15 min at room temperature followed by centrifugation at 4000 *g* for 3 s. The supernatant was collected and mixed with another portion of the beads and incubated as above for three successive times. The bead pellets from each incubation were separately transferred to a 200-μl pipet tip plugged with one layer of C8 empore disks (3M Empore 14-386). The beads were washed two times with 80% acetonitrile (v/v), 2% TFA (v/v) solution. The phosphopeptides were eluted from the beads with two successive 50-µl 40% acetonitrile (v/v) containing 2.5% NH_4_OH (v/v) and then vacuum dried.

#### Isobaric TMT labeling and fractionation

Dried samples obtained after phosphoenrichment were resuspended in 25 µl of 100-mM TEAB and labeled using a TMT 6-plex isobaric label reagent as similarly described above. Once quenched with 1-M Tris-HCl pH 7.5, the labeled samples were combined together, and vacuum dried. The samples were resuspended in 10-mM ammonium formate pH 10.5 and desalted on a C18 resin (Reposil-Pur Basic C18 10 µm, Dr Maisch Gmbh r10.b9.0025) maintained on a 10-µm pore membrane (Mo Bi Tec M2110). The combined samples were then eluted in six fractions of increasing concentration of acetonitrile (7, 10, 12, 17, 22, 50%; v/v) with 3% ammonium formate (v/v). The six fractions were then vacuum dried, washed with 70% acetonitrile, 0.1% formic acid, and kept dried until injection into the mass spectrometer.

#### MS analysis

The six fractions for each combined samples were injected on an Orbitrap Fusion (Thermo Fisher). Each injection was separated on a 50 cm by 75 µm EASY-SprayTM C18 LC column (Thermo Scientific) in a 100-min gradient of solvent A (0.1% formic acid in water) and solvent B (99.9% acetonitrile, 0.1% Formic acid in water) on a Easy LC 1000 system (Thermo Fisher Scientific), coupled with Orbitrap Fusion mass spetrometer (Thermo Fisher Scientific). Survey full scan MS spectra (*m/z* 350–1550) were acquired with a resolution of *r* = 60,000 (at *m/z* 200 Th), an AGC target of 4e5, and a maximum injection time of 100 ms. MS/MS acquisition was acquired in Orbitrap analyzer (AGC target 8e4; HCD 36; *r* = 15,000). A dynamic exclusion was applied using a maximum exclusion list of 500 with one repeat count, repeat, and exclusion duration of 60 s.

#### MS data analysis

Peak lists were generated using Thermo Proteome Discoverer software (v2.1.0, Thermo Fisher Scientific). Spectra were searched using Mascot against target-decoy Mouse Uniprot database. Carbamidomethyl cysteine and TMT 6-plex labeling on N-terminus peptide and Lysine were set as fixed modifications. Phospho S/T/Y, oxidated (M), deamidated (NQ), and acetylated protein N-terminus were set as variable modifications. For peptide assignment, minimal length of six amino acids and maximum three missed cleavages was required, while allowing for maximum 30-ppm mass deviation for MS survey scan and 0.06-Da mass deviation for MS/MS ion fragments, respectively. FDR control was performed on both PSM and peptide level at the level of 0.01 for high and 0.05 for medium confidence peptides. The co-isolation threshold for reporter ion quantification was set at 50%. Protein groups were assembled for downstream data analysis (see Table S3).

### Data normalization, statistical analysis, and graphs

Statistical analysis was performed in R with Limma (v3.42.2) and isobar (v1.19.1) packages [98, 99] to identify differentially regulated proteins while only isobar for phosphorylation sites (phosphopeptides). Mascot DAT files were first parsed with mascotParser2.pl and psx2tab2.pl, and integrated with Mascot MGF files using scripts provided in the isobar package. Samples were normalized to the same median protein intensity across all channel, and average protein ratios between the two experimental conditions (*Mastl*^*FLOX*^ WT and *Mastl*^*NULL*^ KO) across three biological replicates computed using isobar. A noise model for signal intensity is estimated in absence of technical replicates, and observed random protein ratios are fitted with a Cauchy distribution, which are used to estimate the statistical significance of observed variation in protein ratios across the two experimental conditions. Identified phosphorylation sites were analyzed similarly with isobar where the median intensity of phospho-peptide species was normalized to the same value across all channels (samples). Random variation of phospho-peptide ratios is fitted with a Cauchy distribution, and statistical significance of variation observed between experimental conditions computed. Phospho-peptide ratios were then corrected for changes in protein abundance, and statistical significance is recomputed after adjustment of variance. Graphs were created in R with ggplot2 package (v3.2.1) or in Excel.

### In silico kinase prediction

Prediction of the kinase acting on a phosphorylation site was performed using KinomeXplorer [100]. Since KinomeXplorer is human-protein-based, we first mapped the protein IDs of our MS/MS data to human orthologs. After that, it was fed as input to KinomeXplorer. To enable this procedure, we downloaded the KinomeXplorer local version and modified the python script. For Ortholog mapping, Ensembl and HGNC databases were used. Since the kinase predicted by KinomeXplorer are human kinases, we have mapped them to mouse orthologs again. KSEA was performed in R using KSEAapp package (v0.99.0) [101].

### Network visulaization and enrichment analysis

Phosphorylation data were added over a protein–protein interaction network obtained from StringDB for the regulated posphoproteins in Cytoscape (v3.7.2). Enrichment analysis and network visualization were achieved using the ClueGO plugin (v2.5.5) [102] in Cytoscape using mouse annotations from gene ontology biological processes and the Reactome (update: 17 December 2019). Enrichent used a two-side hypergeometric test, corrected with Bonferroni step down method and redundant groups with >60% overlap were merged. Grouping required at least three proteins. Term fusion was allowed for gene ontologies anotations.

## Supplementary information

combined supplementary materials with figures

Table S1

Table S2

Table S3

Table S4

Table S5

Table S6

File S1

## Data Availability

All MS data are available on JPOST repository as JPST000837.
