# Supplementary material for: The Greatwall kinase safeguards the genome integrity by affecting the kinome activity in mitosis
Source: Oncogene. 2020 Sep 25;39(44):6816–40. doi: 10.1038/s41388-020-01470-1 (PMC7605441; doi:10.1038/s41388-020-01470-1)
Supplement: Supplementary file 1 — combined supplementary materials with figures [file 41388_2020_1470_MOESM1_ESM.pdf]

Supplementary materials

Figure S1. Appearance of micronuclei after the loss of Mastl.

Figure S2. Chromosomes breaks in absence of MASTL.

Figure S3. Phosphorylation level, not expression are reproducibly altered after the loss of Mastl.

Figure S4. Network of term enrichment mapping of altered phosphoproteins.

Figure S5. Mastl ablation affects the phosphorylation of CDKs substrates.

Figure S6. Inhibition of NEK2 induces appearance of chromosomal fragments.

Figure S7. Kinase assay of 8 mitotic kinases on peptides with attenuated phosphorylations (1st serie).

Figure S8. Kinase assay of 8 mitotic kinases on peptides with attenuated phosphorylations (2nd serie).

Figure S9. Comparaison of the phosphorylation of 101 substrates by 8 mitotic kinases.

Figure S10. Kinase assay of MASTL on different substrates.

Figure S11. Western blotting of whole cell lysates of transfected HEK293T cells.

Table S1 : Dataframe for quantitative proteome analysis by mass spectrometry.

Table S2 : Dataframe for quantitative phosphoproteome analysis by mass spectrometry.

Table S3 : ClueGO results for Up- and Down-regulated phosphoproteins using reactome and GO_BP terms.

Table S4 : Dataframe for score for the kinase set enrichment analysis using KinomeXplorer scores on the phopshoproteome data.

Table S5 : Dataframe for informations and quantifications of the kinase assays presented in Figure 6A, S7-S9.

Table S6 : List of plasmids and antibodies used in this study.

File S1 : Cytoscape ClueGO file gathering the network of protein-protein interactions from proteins with up- and downregulated phosphorylations;

the network of enriched pathways from the Reactome and gene ontologies - biological processes based on up- and downegulated phosphoproteins. (to be opened with ClueGO app in Cytoscape).

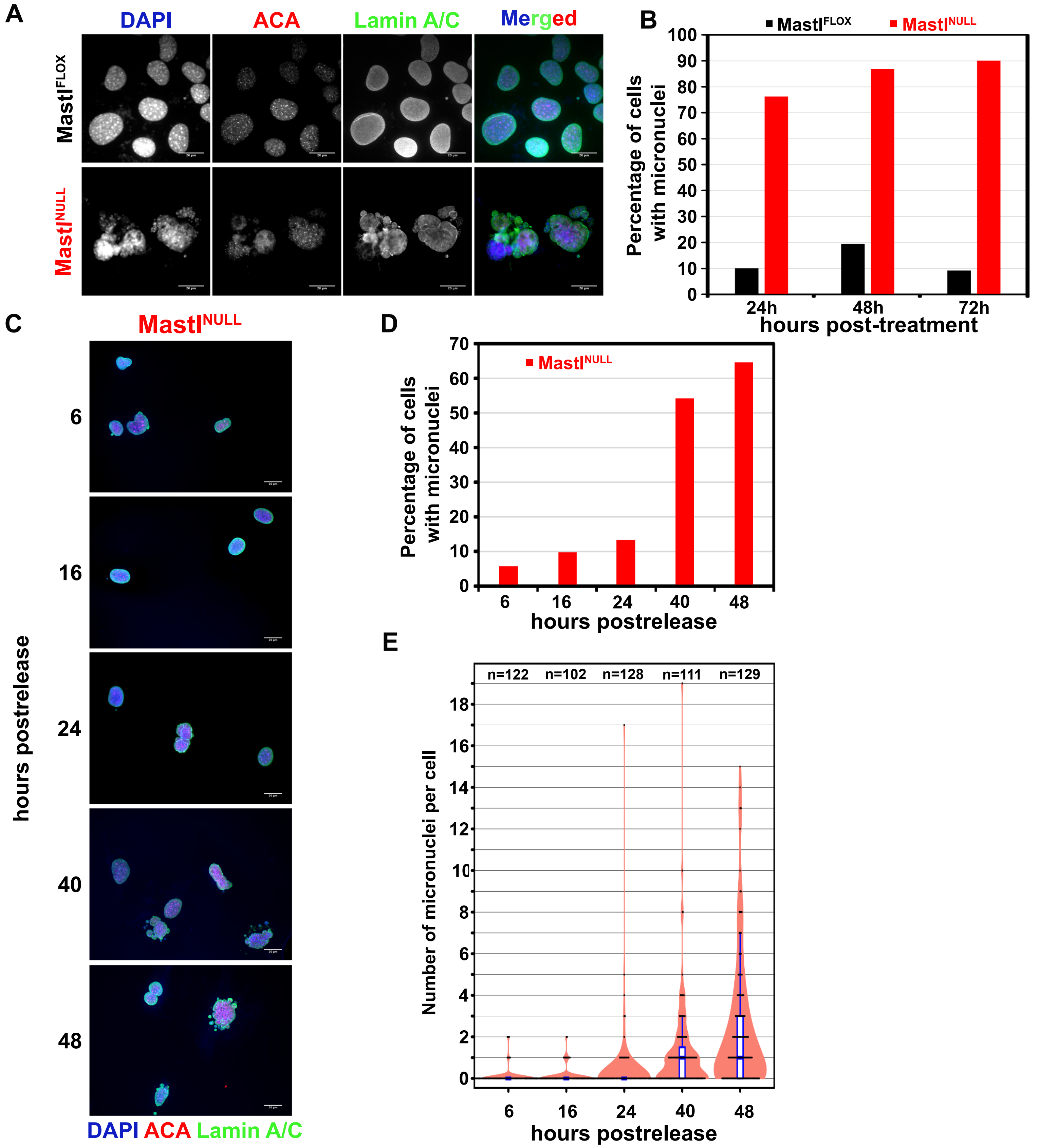

Figure S1. Appearance of micronuclei after the loss of Mastl.

(A, C) Representative immunofluorescence images of (A) Mastl^{FLOX} and Mastl^{NULL} iMEFs collected 72 hours after treatment (EtOH or 4-OHT) or of (C) Mastl^{NULL} iMEFs collected at the indicated time points after release from serum starvation. Cells were immuno-stained with the indicated antibodies.

(B, D) Bar graph of the percentage of cells presenting micronuclei (B) over the time after treatment (EtOH or 4-OHT) or (D) after release from serum starvation.

(E) Violin plot of the distribution of the number of micronuclei per cell over the time after release of cells from serum starvation. n: number of counted cells.

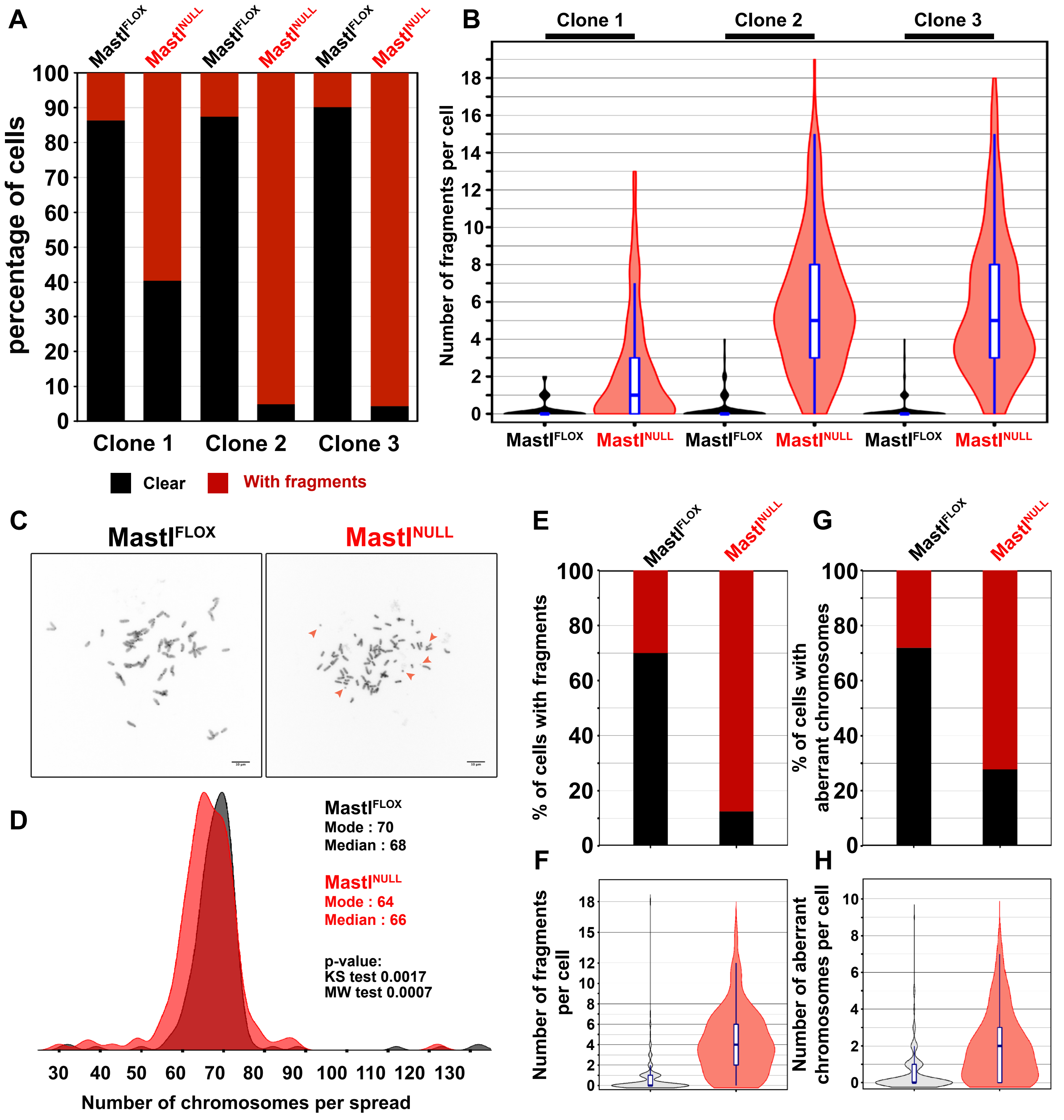

Figure S2. Chromosomes breaks in absence of MASTL

(A) Bar graph of the percentage of cells with or without fragments from 3 separate clones of Mastl^{FLOX} and Mastl^{NULL} primary MEFs.

(B) Violin plots depicting the distribution of micronuclei per cell from the same 3 three pMEF clones as in (A).

(C) Representative images of chromosomes spread from Mastl^{FLOX} and Mastl^{NULL} iMEFs stained with Hoechst.

(D) Scaled distribution of the number of chromosomes per spread for Mastl^{FLOX} and Mastl^{NULL} iMEFs.

KS: Kolmogorov-Smirnov test. MW: Mann-Whitney test.

(E, G) Bar graph of the percentage of cells with or without (E) visible chromosomal fragments or (G) visible aberrant chromosomes.

(F, H) Violin plot with boxplot of the scaled distribution of the number of (F) visible chromosomal fragments or (H) visible aberrant chromosomes per cell.

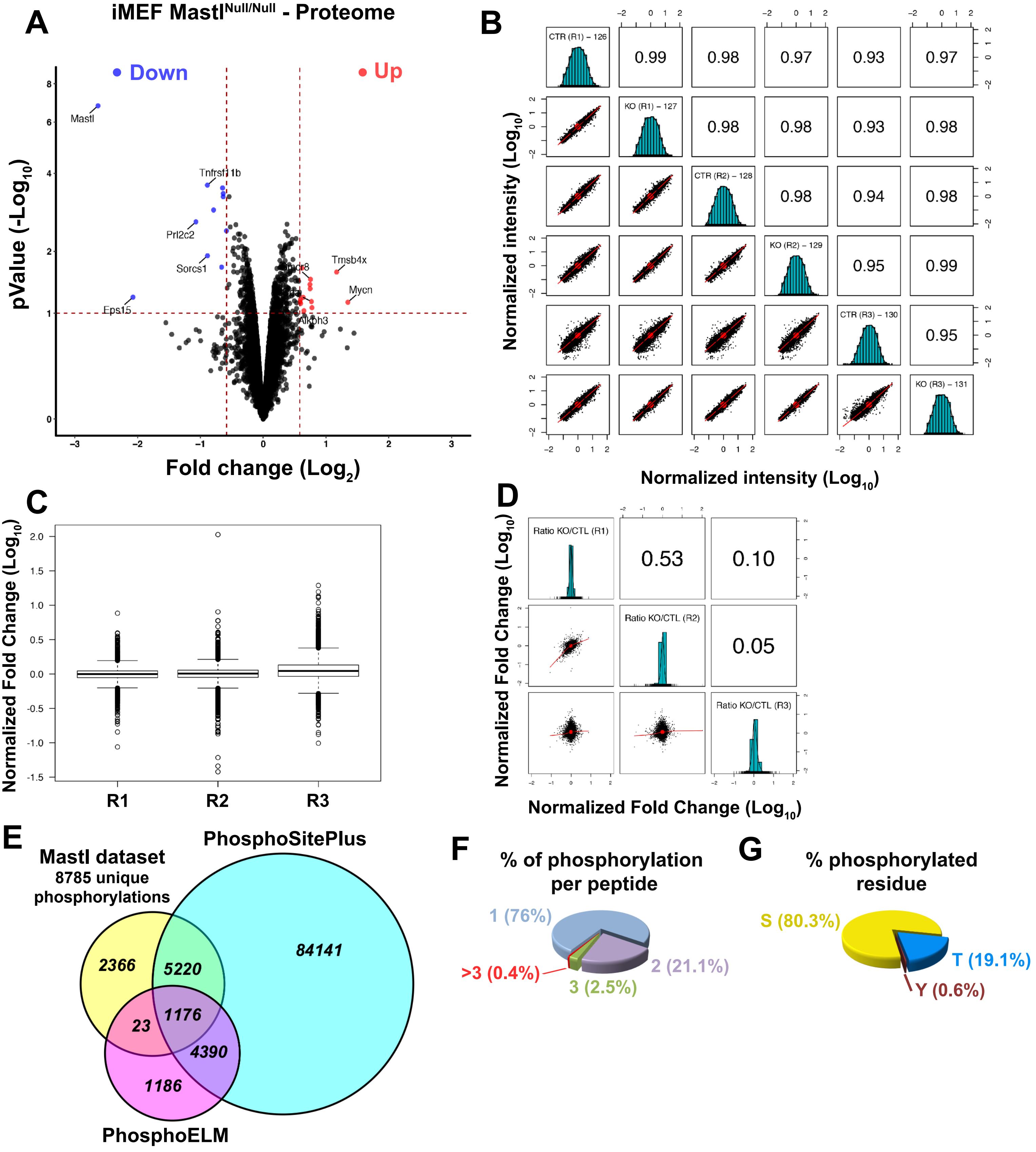

Figure S3. Phosphorylation levels, not expression, are reproducibly altered after the loss of Mastl.

(A) Volcano plot of the fold-change (Mastl^{NULL}/Mastl^{FLOX}) against the p-Value from modified t-test (Limma method) for the quantified proteins.
Red dots: Up-regulated proteins (FC≥1.5 & p-Value≤-0.1). Blue dots: Down-regulated proteins (FC≤0.667 & p-Value≤-0.1). Labels for the 5 most regulated proteins of each category.
(B) Scatterplot of the normalized intensity of phosphopeptides between the 6 samples obtained from 3 biological replicates. Numbers depict the associated Pearson correlation coefficient.
(C) Boxplot of the normalized fold-change of phosphopeptides for each replicate.
(D) Scatterplot of the normalized fold-change of each phosphopeptide between the 3 biological replicates. Numbers depict the associated Pearson correlation coefficient.
(E) Venn diagram of the identified phosphosites from this study in comparison to those reported in PhosphoSitePlus and PhosphoELM databases.
(F, G) Pie charts of (F) the percentage of phosphorylated residues per identified phosphopeptides or (G) the percentage of phosphorylated Serine, Threonine and Tyrosine residues in this study.

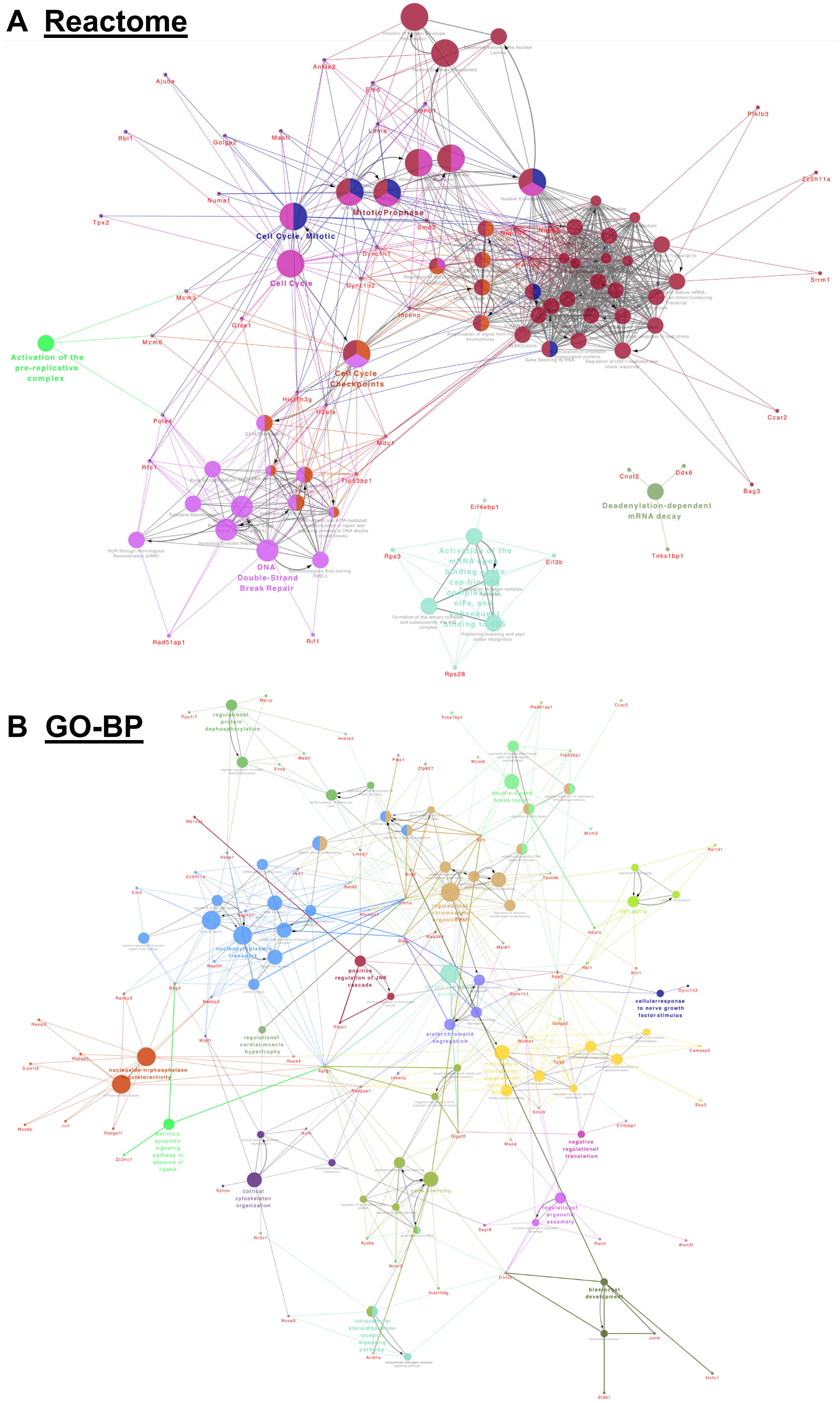

A Reactome
Cell Cycle, Mitotic
Cell Cycle
Mitotic Prophase
Cell Cycle Checkpoints
Activation of the pre-replicative complex
DNA Double-Strand Break Repair
Deadenylation-dependent mRNA decay
Ankle2
Ajuba
Emd
Lmnb1
Lmna
Rbl1
Golga2
Mastl
Numa1
Tpx2
Smc3
Dync1i1
Dync1li2
Mcm3
Mcm6
Gtse1
Incenp
Pole4
Rfc1
Hist1h3g
H2afx
Mdc1
Trp53bp1
Rad51ap1
Rif1
Eif4ebp1
Rps3
Eif3b
Rps28
Cnot2
Ddx6
Tnks1bp1
Bag3
Ccar2
Srrm1
Zc3h11a
Pfkfb3
B GO-BP
regulation of protein dephosphorylation
nucleocytoplasmic transport
positive regulation of JNK cascade
regulation of chromosome organization
cell aging
cellular response to nerve growth factor stimulus
sister chromatid segregation
regulation of cardiac muscle hypertrophy
nucleoside-triphosphatase regulator activity
extrinsic apoptotic signaling pathway in absence of ligand
cortical cytoskeleton organization
gene silencing
negative regulation of translation
regulation of organelle assembly
blastocyst development
intracellular steroid hormone receptor signaling pathway

Figure S4. Network of term enrichment mapping of altered phosphoproteins.

Network of term mapping connected to the annotated proteins based on **(A)** the Reactome or **(B)** the biological processes of gene ontologies.
Term merging indicate cluster of similar terms with different colours.

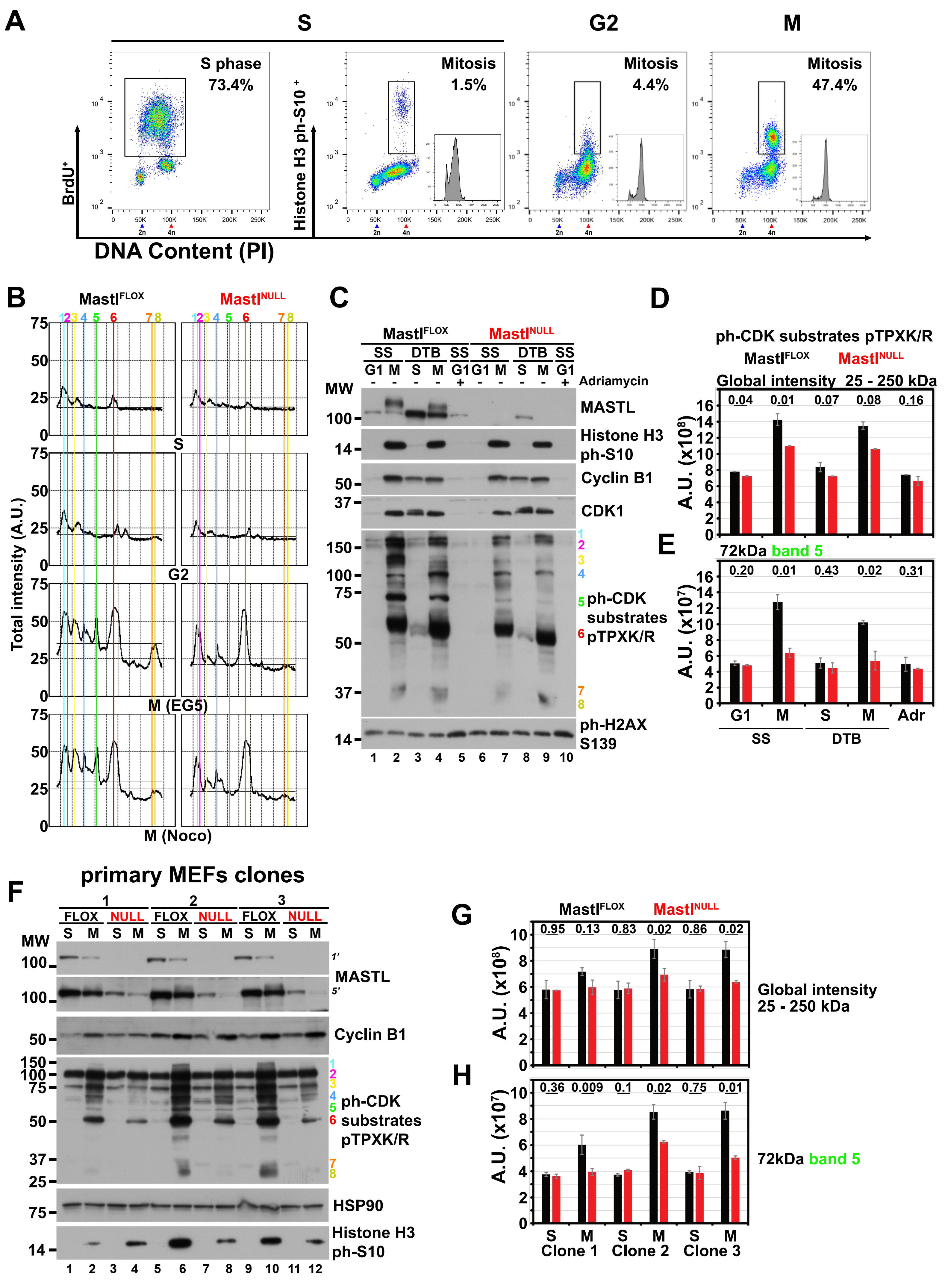

A
S
G2
M
S phase 73.4%
Mitosis 1.5%
Mitosis 4.4%
Mitosis 47.4%
BrdU+
Histone H3 ph-S10+
DNA Content (PI)
2n
4n
B
Mastl FLOX
Mastl NULL
S
G2
M (EG5)
M (Noco)
Total intensity (A.U.)
C
Mastl FLOX
Mastl NULL
SS
DTB
G1 M S M G1 G1 M S M G1
Adriamycin
MW
MASTL
Histone H3 ph-S10
Cyclin B1
CDK1
ph-CDK substrates pTPXK/R
ph-H2AX S139
1 2 3 4 5 6 7 8 9 10
D
ph-CDK substrates pTPXK/R
Global intensity 25 - 250 kDa
0.04 0.01 0.07 0.08 0.16
A.U. (x10^8)
E
72kDa band 5
0.20 0.01 0.43 0.02 0.31
A.U. (x10^7)
G1 M S M Adr
SS
DTB
F
primary MEFs clones
FLOX NULL
S M
MASTL
Cyclin B1
ph-CDK substrates pTPXK/R
HSP90
Histone H3 ph-S10
1 2 3 4 5 6 7 8 9 10 11 12
G
Global intensity 25 - 250 kDa
0.95 0.13 0.83 0.02 0.86 0.02
A.U. (x10^8)
H
72kDa band 5
0.36 0.009 0.1 0.02 0.75 0.01
A.U. (x10^7)
S M S M S M
Clone 1 Clone 2 Clone 3

Figure S5. Mastl ablation affects the phosphorylation of CDKs substrates

(A) FACS scatterplot of iMEFs collected at different phases of the cell cycle and immunostained for BrdU incorporation (S phase) or phospho-Histone H3 on S10 (mitotic cells) with propidium iodide (PI).
(B) Spectral view of the signal intensity given by antibody against ph-CDK substrates pTPXK/R from whole cell lysates of Mastl^{FLOX} and Mastl^{NULL} iMEFs from 25 to 250 kDalton, as shown in Fig. 3D.
Coloured numbers: ph-CDK substrates pTPXK/R. EG5: Eg5 inhibition. Noco: Nocodazole to arrest cells in mitosis.
(C, F) Gel separation of whole cell lvysates of (E) Mastl^{FLOX} or Mastl^{NULL} iMEFs synchronized by serum starvation [SS] or double thymidine block [DTB] or **(H)** three separate primary MEFs as shown in Fig. S2A & B, collected at different cell cycle phases and immunoblotted with antibodies against the indicated proteins (N=1).
MW: Molecular weight ladder. Coloured numbers: ph-CDK substrates pTPXK/R.
(D, E, G, H) Signal quantification of the global signal or of the indicated bands revealed by the antibody against phospho-CDK substrates depicted in (E) and (H), respectively.
A.U. Arbitrary Unit. Error bars depict SD.
pValue obtained from paired t-test on log2 transformed values from separate blots.

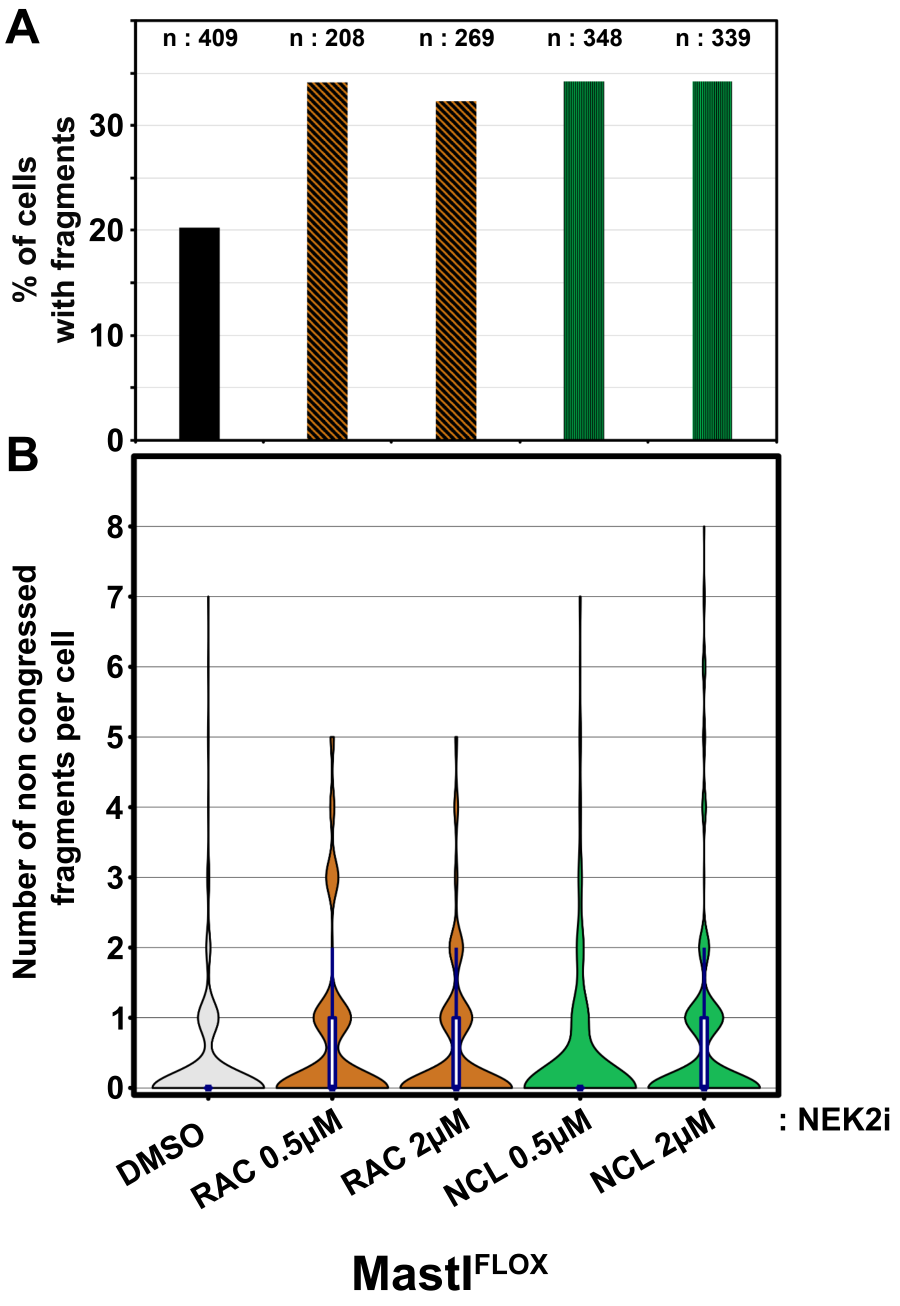

Figure S6.

(A) Bar graph of the percentage of MastlFLOX iMEFs with fragments after treatment with DMSO or a NEK2 inhibitor at the indicated concentration after double thymidine block, arrested and collected in mitosis with nocodazole (N=1).
(B) Violin plots of the distribution of the number of fragments per cell after NEK2 inhibition as shown in (B).
RAC: rac-CCT 250863, NCL: NCL-0001709, n: number of counted cells.

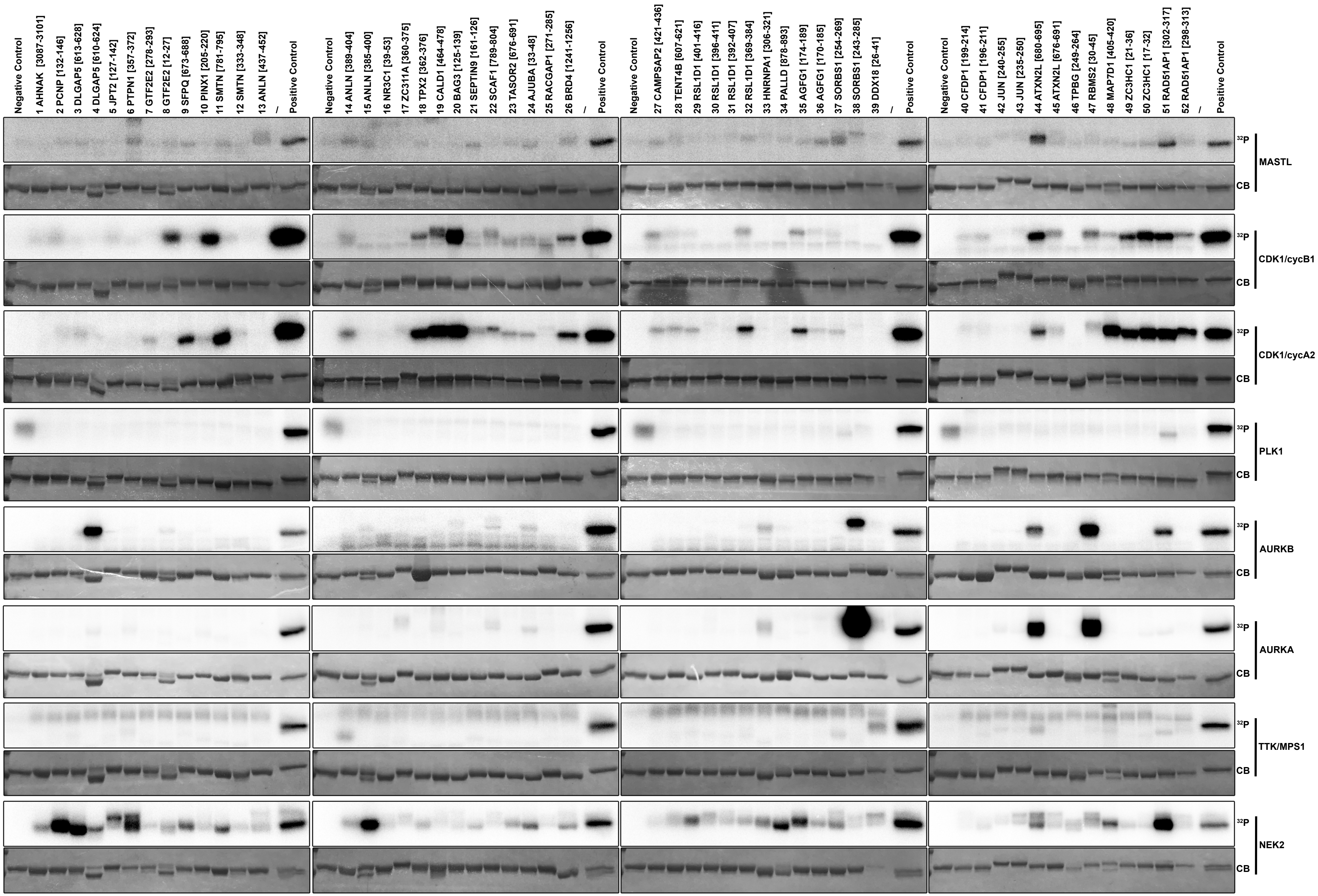
Negative Control
1 AHNAK [3087-3101]
2 PCNP [132-146]
3 DLGAP5 [613-628]
4 DLGAP5 [610-624]
5 JPT2 [127-142]
6 PTPN1 [357-372]
7 GTF2E2 [278-293]
8 GTF2E2 [12-27]
9 SFPQ [673-688]
10 PINX1 [205-220]
11 SMTN [781-795]
12 SMTN [333-348]
13 ANLN [437-452]
/
Positive Control
Negative Control
14 ANLN [389-404]
15 ANLN [385-400]
16 NR3C1 [39-53]
17 ZC3H11A [360-375]
18 TPX2 [362-376]
19 CALD1 [464-478]
20 BAG3 [125-139]
21 SEPTIN9 [161-126]
22 SCAF1 [789-804]
23 TASOR2 [676-691]
24 AJUBA [33-48]
25 RACGAP1 [271-285]
26 BRD4 [1241-1256]
/
Positive Control
Negative Control
27 CAMPSAP2 [421-436]
28 TENT4B [607-621]
29 RSL1D1 [401-416]
30 RSL1D1 [396-411]
31 RSL1D1 [392-407]
32 RSL1D1 [369-384]
33 HNRNPA1 [306-321]
34 PALLD [878-893]
35 AGFG1 [174-189]
36 AGFG1 [170-185]
37 SORBS1 [254-269]
38 SORBS1 [243-285]
39 DDX18 [26-41]
/
Positive Control
Negative Control
40 CFDP1 [199-214]
41 CFDP1 [196-211]
42 JUN [240-255]
43 JUN [235-250]
44 ATXN2L [680-695]
45 ATXN2L [676-691]
46 TPBG [249-264]
47 RBMS2 [30-45]
48 MAP7D1 [405-420]
49 ZC3HC1 [21-36]
50 ZC3HC1 [17-32]
51 RAD51AP1 [302-317]
52 RAD51AP1 [298-313]
/
Positive Control
32P
CB
MASTL
32P
CB
CDK1/cycB1
32P
CB
CDK1/cycA2
32P
CB
PLK1
32P
CB
AURKB
32P
CB
AURKA
32P
CB
TTK/MPS1
32P
CB
NEK2

Figure S7. Kinase assay of 8 mitotic kinases on peptides with attenuated phosphorylations (1st serie).

Representative images of kinase assays of the first 52 peptides of 15 amino acid fused to GST phosphorylated by 8 different kinases or kinase complexes. 32P: Radioactive signal. CB: Coomassie Blue.
Exposure time: MASTL – 18 hours, CDK1/cycB1 – 2 hours, CDK1/cycA2 – 2 hours, PLK1 – 2 hours, AURKB – 30 minutes, AURKA – 18 hours, MPS1/TTK – 1 hour, NEK2 – 1 hour.

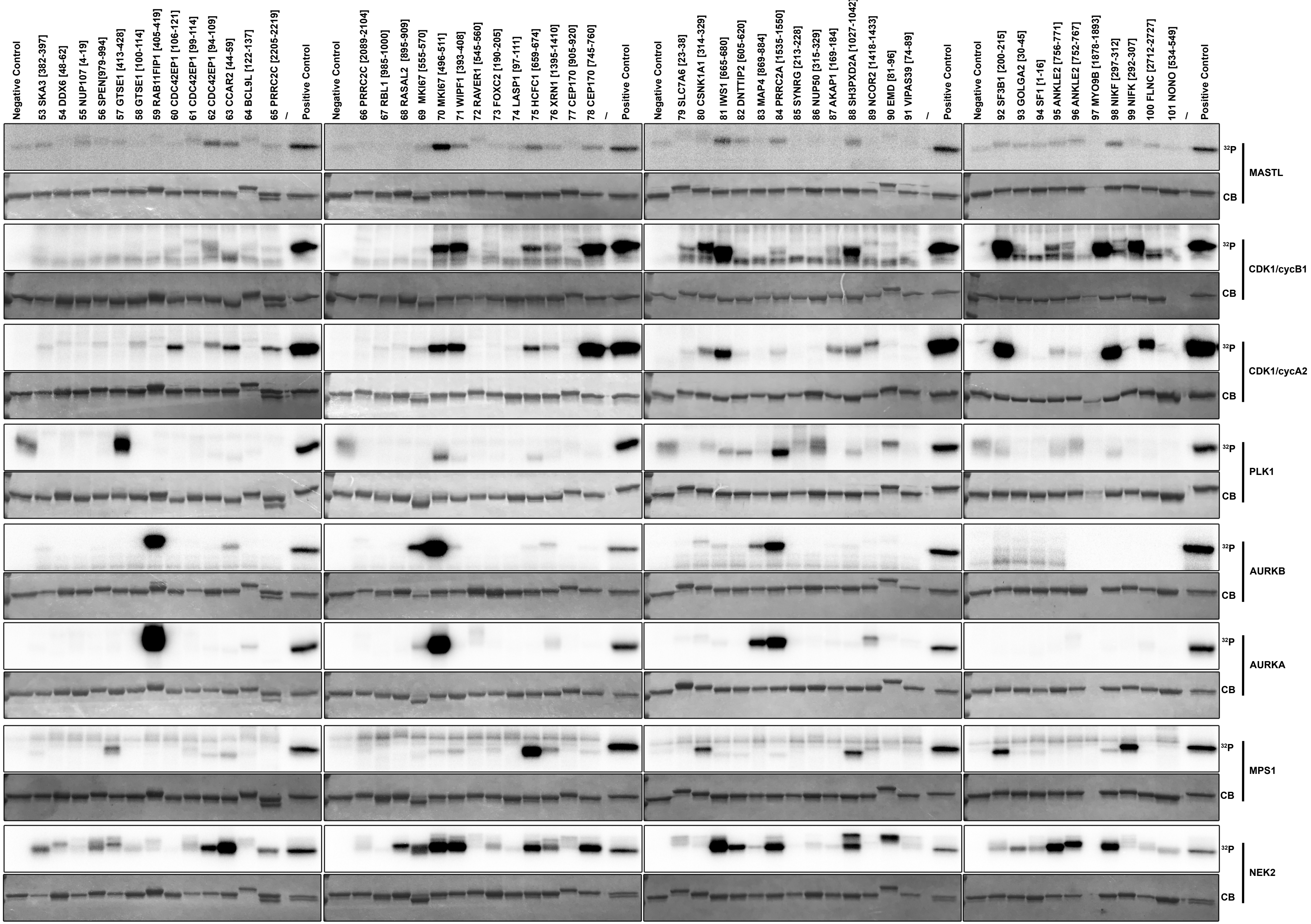
Negative Control
53 SKA3 [382-397]
54 DDX6 [48-62]
55 NUP107 [4-19]
56 SPEN[979-994]
57 GTSE1 [413-428]
58 GTSE1 [100-114]
59 RAB11FIP1 [405-419]
60 CDC42EP1 [106-121]
61 CDC42EP1 [99-114]
62 CDC42EP1 [94-109]
63 CCAR2 [44-59]
64 BCL9L [122-137]
65 PRRC2C [2205-2219]
/
Positive Control
Negative Control
66 PRRC2C [2089-2104]
67 RBL1 [985-1000]
68 RASAL2 [895-909]
69 MKI67 [555-570]
70 MKI67 [496-511]
71 WIPF1 [393-408]
72 RAVER1 [545-560]
73 FOXC2 [190-205]
74 LASP1 [97-111]
75 HCFC1 [659-674]
76 XRN1 [1395-1410]
77 CEP170 [905-920]
78 CEP170 [745-760]
/
Positive Control
Negative Control
79 SLC7A6 [23-38]
80 CSNK1A1 [314-329]
81 IWS1 [665-680]
82 DNTTIP2 [605-620]
83 MAP4 [869-884]
84 PRRC2A [1535-1550]
85 SYNRG [213-228]
86 NUP50 [315-329]
87 AKAP1 [169-184]
88 SH3PXD2A [1027-1042]
89 NCOR2 [1418-1433]
90 EMD [81-96]
91 VIPAS39 [74-89]
/
Positive Control
Negative Control
92 SF3B1 [200-215]
93 GOLGA2 [30-45]
94 SF1 [1-16]
95 ANKLE2 [756-771]
96 ANKLE2 [752-767]
97 MYO9B [1878-1893]
98 NIKF [297-312]
99 NIFK [292-307]
100 FLNC [2712-2727]
101 NONO [534-549]
/
Positive Control
^{32}P
CB
MASTL
^{32}P
CB
CDK1/cycB1
^{32}P
CB
CDK1/cycA2
^{32}P
CB
PLK1
^{32}P
CB
AURKB
^{32}P
CB
AURKA
^{32}P
CB
MPS1
^{32}P
CB
NEK2

Figure S8. Kinase assay of 8 mitotic kinases on peptides with attenuated phosphorylations (2nd serie).

Representative images of kinase assays of the next 49 peptides of 15 amino acids fused to GST phosphorylated by 8 different kinases or kinase complexes.
32P: Radioactive signal. CB: Coomassie Blue.
Exposure time: MASTL – 18 hours, CDK1/cycB1 – 2 hours, CDK1/cycA2 – 2 hours, PLK1 – 2 hours, AURKB – 30 minutes, AURKA – 18 hours, MPS1/TTK – 1 hour, NEK2 – 1 hour.

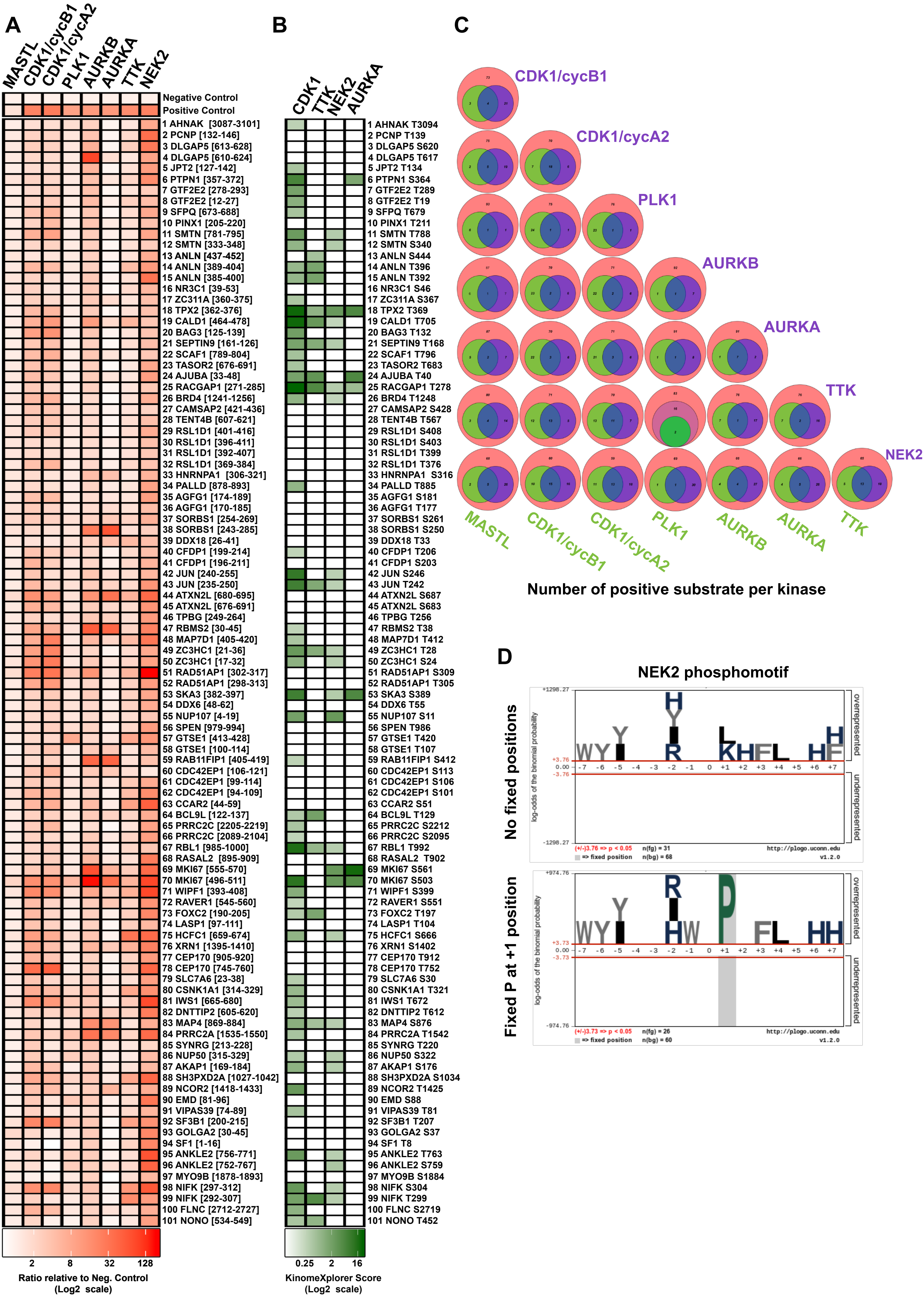
A
MASTL
CDK1/cycB1
CDK1/cycA2
PLK1
AURKB
AURKA
TTK
NEK2
Negative Control
Positive Control
1 AHNAK [3087-3101]
2 PCNP [132-146]
3 DLGAP5 [613-628]
4 DLGAP5 [610-624]
5 JPT2 [127-142]
6 PTPN1 [357-372]
7 GTF2E2 [278-293]
8 GTF2E2 [12-27]
9 SFPQ [673-688]
10 PINX1 [205-220]
11 SMTN [781-795]
12 SMTN [333-348]
13 ANLN [437-452]
14 ANLN [389-404]
15 ANLN [385-400]
16 NR3C1 [39-53]
17 ZC311A [360-375]
18 TPX2 [362-376]
19 CALD1 [464-478]
20 BAG3 [125-139]
21 SEPTIN9 [161-126]
22 SCAF1 [789-804]
23 TASOR2 [676-691]
24 AJUBA [33-48]
25 RACGAP1 [271-285]
26 BRD4 [1241-1256]
27 CAMSAP2 [421-436]
28 TENT4B [607-621]
29 RSL1D1 [401-416]
30 RSL1D1 [396-411]
31 RSL1D1 [392-407]
32 RSL1D1 [369-384]
33 HNRNPA1 [306-321]
34 PALLD [878-893]
35 AGFG1 [174-189]
36 AGFG1 [170-185]
37 SORBS1 [254-269]
38 SORBS1 [243-285]
39 DDX18 [26-41]
40 CFDP1 [199-214]
41 CFDP1 [196-211]
42 JUN [240-255]
43 JUN [235-250]
44 ATXN2L [680-695]
45 ATXN2L [676-691]
46 TPBG [249-264]
47 RBMS2 [30-45]
48 MAP7D1 [405-420]
49 ZC3HC1 [21-36]
50 ZC3HC1 [17-32]
51 RAD51AP1 [302-317]
52 RAD51AP1 [298-313]
53 SKA3 [382-397]
54 DDX6 [48-62]
55 NUP107 [4-19]
56 SPEN [979-994]
57 GTSE1 [413-428]
58 GTSE1 [100-114]
59 RAB11FIP1 [405-419]
60 CDC42EP1 [106-121]
61 CDC42EP1 [99-114]
62 CDC42EP1 [94-109]
63 CCAR2 [44-59]
64 BCL9L [122-137]
65 PRRC2C [2205-2219]
66 PRRC2C [2089-2104]
67 RBL1 [985-1000]
68 RASAL2 [895-909]
69 MKI67 [555-570]
70 MKI67 [496-511]
71 WIPF1 [393-408]
72 RAVER1 [545-560]
73 FOXC2 [190-205]
74 LASP1 [97-111]
75 HCFC1 [659-674]
76 XRN1 [1395-1410]
77 CEP170 [905-920]
78 CEP170 [745-760]
79 SLC7A6 [23-38]
80 CSNK1A1 [314-329]
81 IWS1 [665-680]
82 DNTTIP2 [605-620]
83 MAP4 [869-884]
84 PRRC2A [1535-1550]
85 SYNRG [213-228]
86 NUP50 [315-329]
87 AKAP1 [169-184]
88 SH3PXD2A [1027-1042]
89 NCOR2 [1418-1433]
90 EMD [81-96]
91 VIPAS39 [74-89]
92 SF3B1 [200-215]
93 GOLGA2 [30-45]
94 SF1 [1-16]
95 ANKLE2 [756-771]
96 ANKLE2 [752-767]
97 MYO9B [1878-1893]
98 NIFK [297-312]
99 NIFK [292-307]
100 FLNC [2712-2727]
101 NONO [534-549]
2 8 32 128
Ratio relative to Neg. Control
(Log2 scale)
B
CDK1
TTK
NEK2
AURKA
1 AHNAK T3094
2 PCNP T139
3 DLGAP5 S620
4 DLGAP5 T617
5 JPT2 T134
6 PTPN1 S364
7 GTF2E2 T289
8 GTF2E2 T19
9 SFPQ T679
10 PINX1 T211
11 SMTN T788
12 SMTN S340
13 ANLN S444
14 ANLN T396
15 ANLN T392
16 NR3C1 S46
17 ZC311A S367
18 TPX2 T369
19 CALD1 T705
20 BAG3 T132
21 SEPTIN9 T168
22 SCAF1 T796
23 TASOR2 T683
24 AJUBA T40
25 RACGAP1 T278
26 BRD4 T1248
27 CAMSAP2 S428
28 TENT4B T567
29 RSL1D1 S408
30 RSL1D1 S403
31 RSL1D1 T399
32 RSL1D1 T376
33 HNRNPA1 S316
34 PALLD T885
35 AGFG1 S181
36 AGFG1 T177
37 SORBS1 S261
38 SORBS1 S250
39 DDX18 T33
40 CFDP1 T206
41 CFDP1 S203
42 JUN S246
43 JUN T242
44 ATXN2L S687
45 ATXN2L S683
46 TPBG T256
47 RBMS2 T38
48 MAP7D1 T412
49 ZC3HC1 T28
50 ZC3HC1 S24
51 RAD51AP1 S309
52 RAD51AP1 T305
53 SKA3 S389
54 DDX6 T55
55 NUP107 S11
56 SPEN T986
57 GTSE1 T420
58 GTSE1 T107
59 RAB11FIP1 S412
60 CDC42EP1 S113
61 CDC42EP1 S106
62 CDC42EP1 S101
63 CCAR2 S51
64 BCL9L T129
65 PRRC2C S2212
66 PRRC2C S2095
67 RBL1 T992
68 RASAL2 T902
69 MKI67 S561
70 MKI67 S503
71 WIPF1 S399
72 RAVER1 S551
73 FOXC2 T197
74 LASP1 T104
75 HCFC1 S666
76 XRN1 S1402
77 CEP170 T912
78 CEP170 T752
79 SLC7A6 S30
80 CSNK1A1 T321
81 IWS1 T672
82 DNTTIP2 T612
83 MAP4 S876
84 PRRC2A T1542
85 SYNRG T220
86 NUP50 S322
87 AKAP1 S176
88 SH3PXD2A S1034
89 NCOR2 T1425
90 EMD S88
91 VIPAS39 T81
92 SF3B1 T207
93 GOLGA2 S37
94 SF1 T8
95 ANKLE2 T763
96 ANKLE2 S759
97 MYO9B S1884
98 NIFK S304
99 NIFK T299
100 FLNC S2719
101 NONO T452
0.25 2 16
KinomeXplorer Score
(Log2 scale)
C
CDK1/cycB1
CDK1/cycA2
PLK1
AURKB
AURKA
TTK
NEK2
MASTL
CDK1/cycB1
CDK1/cycA2
PLK1
AURKB
AURKA
TTK
Number of positive substrate per kinase
D
NEK2 phosphomotif
No fixed positions
+1298.27
-1298.27
+3.76
0.00
-3.76
-7 -6 -5 -4 -3 -2 -1 0 +1 +2 +3 +4 +5 +6 +7
log-odds of the binomial probability
overrepresented
underrepresented
(+/-)3.76 => p < 0.05
=> fixed position
n(fg) = 31
n(bg) = 68
http://plogo.uconn.edu
v1.2.0
Fixed P at +1 position
+974.76
-974.76
+3.73
0.00
-3.73
-7 -6 -5 -4 -3 -2 -1 0 +1 +2 +3 +4 +5 +6 +7
log-odds of the binomial probability
overrepresented
underrepresented
(+/-)3.73 => p < 0.05
=> fixed position
n(fg) = 26
n(bg) = 60
http://plogo.uconn.edu
v1.2.0

Figure S9. Comparaison of the phosphorylation of 101 substrates by 8 mitotic kinases.

(A) Heatmap of the mean ratio of intensity (tested GST fused 15 amino acid peptides/negative control) obtained from three replicates of each kinase assay using 8 different kinases on 101 substrates (Fig. S7-8, Table S5).
(B) Heatmap of the KinomeXplorer score for the indicated phosphosites as predicted for four kinases.
(C) Venn graphs of phosphorylated substrates between two kinases.
(D) Motif probability [pLogo, (103)] based on NEK2 phosphorylated substrates using no fixed positions or with a fixed proline (in 83% of phosphorylated substrates) at +1 position.

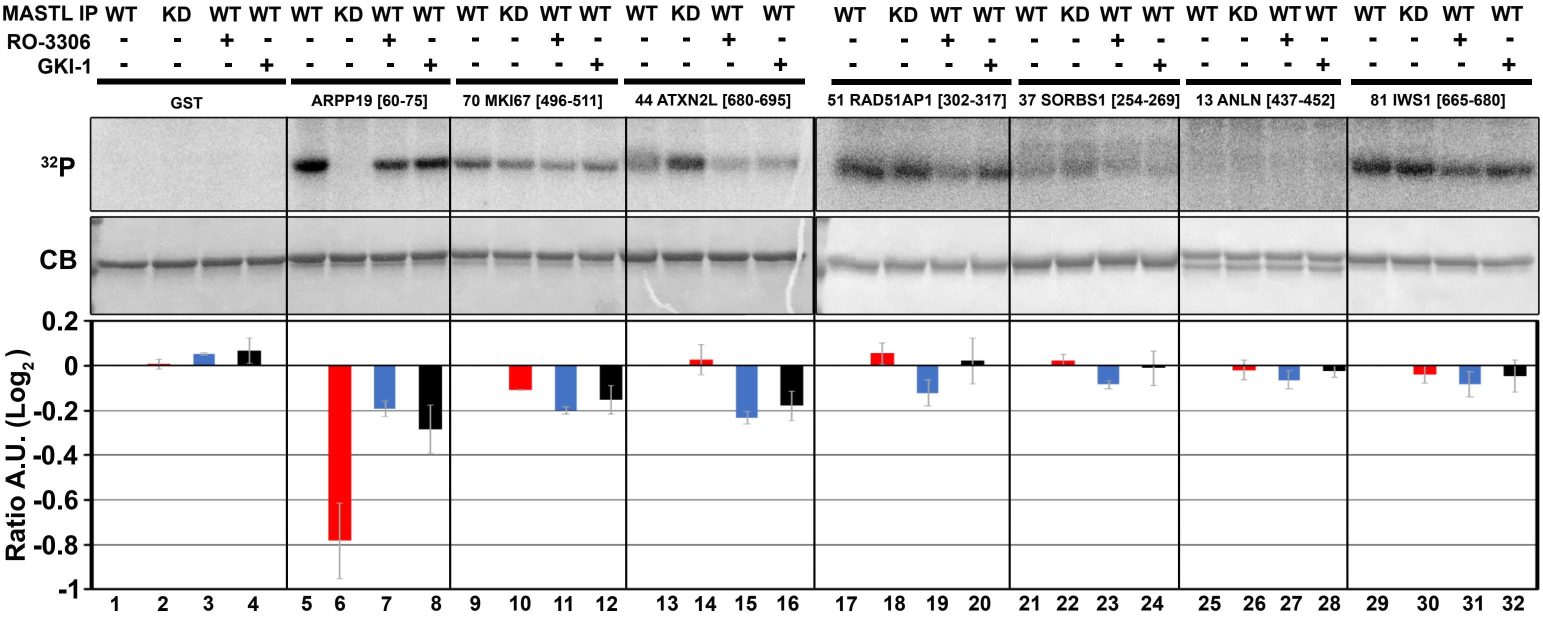

Figure S10. Kinase assay of MASTL on different substrates.

Representative images of kinase assays of the 6 most phosphorylated peptides of 15 amino acid fused to GST phosphorylated by wild-type (WT) or kinase dead (KD) MASTL in the absence or presence of CDK1 inhibition (RO-3306) or Greatwall kinase inhibitor 1 (GK1-1).
Bar graph of the averaged ratio (condition/untreated WT MASTL) of three separate replicates of the kinase assays.
32P: Radioactive signal. CB: Coomassie Blue.

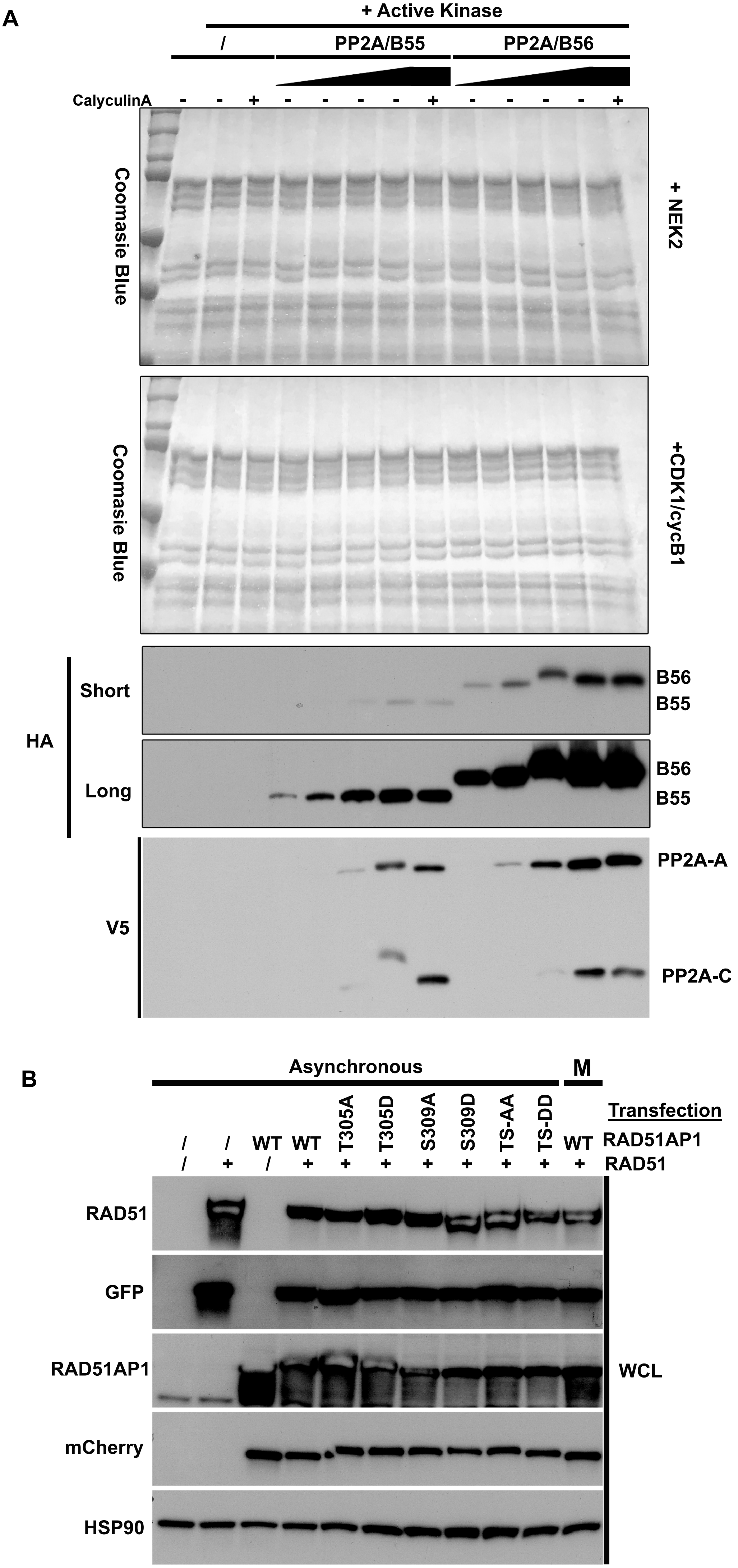
A
+ Active Kinase
/
PP2A/B55
PP2A/B56
CalyculinA
Coomasie Blue
+ NEK2
Coomasie Blue
+CDK1/cycB1
HA
Short
Long
B56
B55
B56
B55
V5
PP2A-A
PP2A-C
B
Asynchronous
M
Transfection
RAD51AP1
RAD51
/ / WT WT T305A T305D S309A S309D TS-AA TS-DD WT
/ + / + + + + + + + +
RAD51
GFP
RAD51AP1
mCherry
HSP90
WCL

Figure S11. Western blotting of whole cell lysates of transfected HEK293T cells.

(A) Gel separation of the phosphatase assays presented in Fig. 5C and stained with Coomassie blue for the substrates used or immunoblotted for the immunoprecipitated subunits of PP2A complexes.
(B) Gel separation of whole cell lysates from transiently transfected asynchronous or mitotic (M) 293T cells immunoblotted with antibodies against the indicated proteins.
